# New metrics for multiple testing with correlated outcomes

**DOI:** 10.3389/fams.2023.1151314

**Published:** 2023-05-01

**Authors:** Maya B. Mathur, Tyler J. VanderWeele

**Affiliations:** 1 Quantitative Sciences Unit and Department of Pediatrics, Stanford University, Palo Alto, CA, United States; 2 Department of Epidemiology, Harvard T. H. Chan School of Public Health, Boston, MA, United States

**Keywords:** multiplicity, Type I error, bootstrap, resampling, familywise error rate, multiverse

## Abstract

When investigators test multiple outcomes or fit different model specifications to the same dataset, as in multiverse analyses, the resulting test statistics may be correlated. We propose new multiple-testing metrics that compare the observed number of hypothesis test rejections $\left(\hat{\theta }\right)$ at an unpenalized α-level to the distribution of rejections that would be expected if all tested null hypotheses held (the “global null”). Specifically, we propose reporting a “null interval” for the number of α-level rejections expected to occur in 95% of samples under the global null, the difference between $\hat{\theta }$ and the upper limit of the null interval (the “excess hits”), and a one-sided joint test based on $\hat{\theta }$ of the global null. For estimation, we describe resampling algorithms that asymptotically recover the sampling distribution under the global null. These methods accommodate arbitrarily correlated test statistics and do not require high-dimensional analyses, though they also accommodate such analyses. In a simulation study, we assess properties of the proposed metrics under varying correlation structures as well as their power for outcome-wide inference relative to existing methods for controlling familywise error rate. We recommend reporting our proposed metrics along with appropriate measures of effect size for all tests. We provide an R package, NRejections. Ultimately, existing procedures for multiple hypothesis testing typically penalize inference in each test, which is useful to temper interpretation of individual findings; yet on their own, these procedures do not fully characterize global evidence strength *across* the multiple tests. Our new metrics help remedy this limitation.

## 1. Introduction

In studies testing multiple hypotheses, the problem of inflated Type I error rates is usually handled, if at all, through procedures that preserve familywise error rate (FWER) or false discovery rate (FDR) by penalizing individual p-values or critical values. These procedures can be valuable for individually correcting inference for each hypothesis test. However, as standalone reporting methods, they may provide incomplete insight into the overall strength of evidence across tests. For example, if individual hypothesis tests of the associations between a single exposure of interest and 40 outcome measures result in a total of 10 rejections at an uncorrected α=0.05 and result in 1 rejection at a Bonferroni-corrected α≈0.001, how strong is the overall evidence supporting associations between the exposure and the outcomes, considered jointly? Given only the information typically reported in corrected or uncorrected multiple tests, such questions can be hard to answer.

Intuitive speculation about overall evidence strength becomes especially challenging when the hypothesis tests are correlated, which is typically the case when related research questions are considered or in “outcome-wide” analyses that assess associations between a single exposure and a number of outcomes [1]. Correlated hypothesis tests can also arise when investigators re-analyze a single dataset using numerous different model specifications as a sensitivity analysis, an approach that has rapidly gained traction in the social sciences [2]. Indeed, as we will illustrate, the results of a given set of individual tests (whether multiplicity-corrected or not) may be strongly suggestive of at least some genuine effects if the tests are independent, but may be entirely consistent with chance (in a manner we will formalize) if the tests are correlated. In practice, the correlation structure of the tests is usually unknown, further impeding intuitive assessment.

We therefore aim to supplement existing multiple-testing procedures (e.g., [3–7]) with simple metrics that directly characterize overall evidence strength while accommodating arbitrarily correlated test statistics. These metrics focus on the total number of hypothesis test rejections at an arbitrary α level (such as the usual, uncorrected α=0.05). First, we propose reporting a *null interval* representing a plausible range for the total number of rejections in 95% of samples that would occur if all null hypotheses were true (a scenario we call the “global null”), along with the difference between the number of observed rejections and the upper interval limit (the *excess hits*). For example, if we reject 10 of 40 hypotheses at α=0.05, we might be tempted to conclude intuitively that this is “more” than the expected 0.05 × 40 = 2 rejections. However, if the null interval is [0, 11], accounting for correlation between the tests, this would suggest little evidence overall for genuine associations across the 40 tests. In contrast, if we instead reject 18 tests under the same correlation structure, the null interval indicates that we have observed 7 excess hits beyond the number that would be expected in 95% of samples generated under the global null, which is suggestive of strong overall evidence that at least some of the tested associations are present. Additionally, we propose using the number of rejections to conduct a one-sided *global test* of the global null, whose p-value represents the probability, in samples generated under the global null, of observing at least as many α-level rejections as were actually observed.

Although standard methods for FWER control are not explicitly designed to characterize overall evidence strength, they could in principle be repurposed into a global test. That is, letting $\alpha_{W}$ denote the level of familywise inference, rejection of at least one test with inference corrected to preserve a familywise $\alpha_{W}=0.05$ implies rejection of the global null at $\alpha_{W}=0.05$. Several existing methods strongly control FWER in hypothesis tests with unknown correlation structure and could therefore be suitable for a global test. The most widespread approaches are the classical one-step Bonferroni correction [3] and its uniformly more powerful successor, the step-down Holm method [4], both of which can be computed in closed form. By avoiding specifying or estimating the correlation structure, these naïve methods accommodate even worst-case correlation structures but can yield conservative inference. Other closed-form methods achieve better power by assuming independence [e.g., various procedures based on [8]’s inequality] or known logical dependencies between tests (e.g., [9]) but can produce anticonservative inference if these assumptions are violated [10]. We focus here on methods, detailed in Section 2, that avoid such assumptions by empirically estimating the correlation structure via resampling [5–7]. Related methods control FDR rather than FWER (e.g., [6, 11]), but because FDR control does not appear to have a direct relationship with the null interval and excess hits metrics discussed in the present paper, we do not further consider these methods. Alternative approaches are designed for a large number of hypothesis tests, as in high-dimensional genetic studies (e.g., [12–14]); however, because correlated hypothesis tests can be particularly problematic in traditional low-dimensional settings [15], we aim to provide methods that apply regardless of the number of tests.

In this paper, we first derive assumptions for the asymptotic validity of a resampling-based null interval, the corresponding excess hits, and a global test of the number of rejections, and we describe specific resampling algorithms fulfilling these assumptions. Heuristically, the resampling algorithm must ensure that the distribution of test statistics in the resamples converges to the distribution they would would have under the global null. Critically, this must hold even when the observed data were instead generated under an alternative hypothesis. For example, we consider the case of fitting an ordinary least squares (OLS) multiple regression model to each of several outcomes, using the same design matrix each time. In this setting, we show that one valid resampling algorithm is to fix the covariates for all observations while setting the resampled “outcomes” equal to the fitted values plus a vector of residuals resampled with replacement. As we formalize, this resampling algorithm enforces the global null (even if the observed data were generated under an alternative), while also preserving the correlations between the outcomes and the adjusted covariates. We describe why several alternative, intuitive resampling approaches in fact do not have these properties, and hence may not result in correct global inference. Second, we conduct a simulation study in which we: (1) compare the null interval to the observed number of rejections for varying effect sizes; and (2) assess the relative power of global tests and the number of rejected null hypotheses at a familywise-controlled $\alpha_{W}=0.05$ using the number of rejections or derived from existing FWER-control methods, as discussed above. To our knowledge, prior simulation studies of existing FWER methods have not reported on their performance as global tests [6]. We illustrate application of our proposed metrics through an applied example.

## 2. Existing resampling-based multiplicity corrections

Table 1 summarizes existing methods that strongly control FWER for arbitrarily correlated tests. Westfall and Young [5] propose resampling algorithms that resemble the standard bootstrap, but that modify the data in order to enforce the global null (an approach similar to what we will adopt). For example, suppose we conduct one-sample t-tests on the potentially correlated variables $\left(Y_{1},\cdots ,Y_{W}\right)$ with the global null stating that $E\left[Y_{w}\right]=0\;\forall \;w\in \{1,\cdots ,W\}$. To resample under the global null, the observed data could first be centered by their respective sample means, then resampled with replacement [5]. Thus, in the resampled datasets, the global null holds regardless of the true parameters $\left(E\left[Y_{1}\right],\cdots ,E\left[Y_{W}\right]\right)$ underlying the original sample. Then, Westfall and Young [5]’s one-step “minP” method and uniformly more powerful step-down variant (here termed “Wstep”) adjust the observed p-values using quantiles of the distribution of p-values calculated in the resamples.

Other FWER methods use parametric resampling approaches that do not enforce the global null in the resampled data, but rather that generate datasets resembling the original data [6, 7]. Essentially invoking the duality of hypothesis tests and confidence intervals, the resampled test statistics are then centered by their estimated values in the observed data in order to recover the null distribution; other related methods showed less favorable performance in prior simulations, so we do not further consider them here [6, 11].

This latter class of resampling approaches obviates a key assumption used to simplify computation in the minP and Wstep approaches. This “subset pivotality” assumption has been discussed at length elsewhere (e.g., [5, 7, 16, 17]); it states that for any subset K of hypotheses being tested, if all null hypotheses in K hold, then the joint distribution of test statistics in K is the same regardless of the truth or falsehood of all hypotheses not in K [16] (See Definition 1 below for a more formal definition.). The motivation for subset pivotality is that strong FWER-control methods that empirically estimate the correlation structure must control FWER not only when the global null holds, but also for any configuration of true and false null hypotheses. Although it might appear that resamples would therefore need to be generated under every such configuration, Westfall and Young [5]’s methods circumvent this problem, requiring only one set of resamples under the global null, by invoking subset pivotality.

Subset pivotality can fail, for example, when testing pairwise correlations of three variables, X, Y, and Z [Romano and Wolf [7]’s Example 4.1]. In this setting, the joint distribution of the statistics ${\hat{\rho }}_{XY}$ and ${\hat{\rho }}_{XZ}$ when a particular subset K of the null hypotheses hold, namely $\rho_{XY}=\rho_{XZ}=0$, depends on $\rho_{YZ}$ and hence on the truth or falsehood of a hypothesis not in K [7]. Thus, under [5]’s resampling approach, ${\hat{\rho }}_{XY}^{\left(j\right)}$ and ${\hat{\rho }}_{XZ}^{\left(j\right)}$ would be correctly centered at 0, but they would be independent because the global null is enforced. In contrast, under [6]’s resampling approach, ${\hat{\rho }}_{XY}^{\left(j\right)}$ and ${\hat{\rho }}_{XZ}^{\left(j\right)}$ would likewise be centered at 0 because they would have been centered by the sample estimates ${\hat{\rho }}_{XY}$ and ${\hat{\rho }}_{XZ}$, but they would also be correlated to an extent determined by $\rho_{YZ}$. In turn, the distribution of the maximum test statistic depends on the joint distribution of $\left({\hat{\rho }}_{XY},{\hat{\rho }}_{XZ}\right)$. Importantly, subset pivotality will *not* be required for our proposed null interval and global test: unlike FWER-control methods, our proposed methods concern only the global null, and thus even when subset pivotality does not hold, it is sufficient to estimate via resampling the single sampling distribution of the test statistics under the global null. We will, however, describe a repeated-sampling interpretation of the excess hits that does require subset pivotality to hold. To build upon these existing methods by directly characterizing global evidence strength, we now provide a running example for the setting of linear regression, and then develop theory underlying our proposed metrics.

## 3. Example: valid residual resampling for linear regression

To fix concepts, we first consider the design of valid resampling algorithms for the familiar setting of ordinary least squares (OLS) multiple regression. In subsequent sections, we will give formal conditions for a resampling algorithm to be valid for general settings not restricted to OLS models. As we describe in the Section 9, the practical design of resampling algorithms that fulfill those conditions remains an open problem for many models, such as generalized linear models (GLMs) with non-identity link functions, but the present theory may help stimulate further research on this. Nevertheless, the class of OLS models does subsume a number of common statistical tests, including t-tests and analysis of variance, so we take this as a useful starting point.

Assume that each of W outcome variables, $\left(Y_{1},\cdots ,Y_{W}\right)$, is regressed on the same design matrix, $X\in {\mathbb{R}}^{N\times p}$, comprising an intercept term denoted $X_{1}$ (such that the residuals have mean 0), a single exposure of interest (taken without loss of generality to be $X_{2}$), and the adjusted covariates $\left(X_{3},\cdots ,X_{C}\right)$. Assume all covariates besides the intercept are mean-centered. Thus, the dataset Z contains a random vector $\left(1,X_{n2},\cdots ,X_{nC},Y_{n1},\cdots ,Y_{nW}\right)$ for each subject n. Let $\epsilon_{w}=\left(\epsilon_{1w},\cdots ,\epsilon_{Nw}\right)$ denote the N-vector of true errors for the $w^{th}$ regression such that $\epsilon_{nw}∼N\left(0,\sigma_{w}^{2}\right)$. Let ${\hat{\epsilon }}_{w}$ be its estimated counterpart (the residuals). Let $\sigma_{w}^{2}=E\left[\epsilon_{nw}^{2}|X\right]$ as usual, and assume $\sigma_{w}^{2}<\infty$. Let ${\hat{\gamma }}_{iw}$ be the $i^{th}$ coefficient estimate in the $w^{th}$ regression, and denote the coefficient for the exposure of interest in the $w^{th}$ regression model as ${\hat{\beta }}_{w}={\hat{\gamma }}_{2w}$. The W models are then:

(1) $$\;Y_{n1}=\gamma_{11}+\beta_{1}X_{n2}+\sum_{j=3}^{C}\gamma_{j1}X_{nj}+\epsilon_{n1}\;\;\vdots \;Y_{nW}=\gamma_{1W}+\beta_{W}X_{n2}+\sum_{j=3}^{C}\gamma_{jW}X_{nj}+\epsilon_{nW}$$

Let $\beta^{W}=\left(\beta_{1},\cdots ,\beta_{W}\right)$ be a W-vector containing, for each regression model, the single coefficient of interest, and let ${\hat{\beta }}^{W}$ and ${\hat{\beta }}^{W(j)}$ denote its sample estimate in the original dataset and in resampled dataset j, respectively. Suppose without loss of generality that the null hypotheses of interest are $H_{0w}:\beta_{w}=0$. Letting hats denote the usual OLS estimates obtained from the original sample, the usual test statistics in the original sample are $T=\left(\frac{{\hat{\beta }}_{1}}{{\hat{\sigma }}_{1}{\left(X^{\prime }X\right)}^{-1/2}},\cdots ,\frac{{\hat{\beta }}_{W}}{{\hat{\sigma }}_{W}{\left(X^{\prime }X\right)}^{-1/2}}\right)$; their unobservable counterparts centered to reflect the global null are $T^{0}=\left(\frac{{\hat{\beta }}_{1}-\beta_{1}}{{\hat{\sigma }}_{1}{\left(X^{\prime }X\right)}^{-1/2}},\cdots ,\frac{{\hat{\beta }}_{W}-\beta_{W}}{{\hat{\sigma }}_{W}{\left(X^{\prime }X\right)}^{-1/2}}\right)$.

Algorithm 1. **A valid resampling algorithm for OLS.**

Generalizing from [18], a parametric resampling algorithm that is valid to recover the distribution of test statistics under the global null is to first fix the covariates $\left(X_{1},\cdots ,X_{C}\right)$ for all observations while setting the resampled “outcomes” equal to the fitted values plus a vector of residuals resampled with replacement. That is, letting $n^{\prime }$ denote an observation sampled with replacement, the resampled variables for observation n are:

$$X_{n1}^{\left(j\right)}:=X_{n1}\;Y_{n1}^{\left(j\right)}:={\hat{Y}}_{n1}+\left({\hat{Y}}_{n^{\prime }1}-Y_{n^{\prime }1}\right)\;\;\vdots \;\vdots \;X_{nC}^{\left(j\right)}:=X_{nC}\;Y_{nW}^{\left(j\right)}:={\hat{Y}}_{nW}+\left({\hat{Y}}_{n^{\prime }W}-Y_{n^{\prime }W}\right)$$

Then each test statistic in the resamples is computed using ${\tilde{H}}_{0w}:\beta_{w}={\hat{\beta }}_{w}$ in order to recover the null sampling distribution [19]. That is:

(2) $$T^{\left(j\right)}=\left(\frac{{\hat{\beta }}_{1}^{\left(j\right)}-{\hat{\beta }}_{1}}{{\hat{\sigma }}_{1}^{\left(j\right)}{\left(X^{\prime }X\right)}^{-1/2}},\cdots ,\frac{{\hat{\beta }}_{W}^{\left(j\right)}-{\hat{\beta }}_{W}}{{\hat{\sigma }}_{W}^{\left(j\right)}{\left(X^{\prime }X\right)}^{-1/2}}\right)$$

We show in Supplementary material that this resampling algorithm is valid, as formalized in Section 5 below, because the distribution of each ${\hat{\beta }}_{w}-\beta_{w}={\left(X^{\prime }X\right)}^{-1}X^{\prime }\epsilon_{w}$ (in the original sample) depends only on the true error distribution and not on $\beta_{w}$, so the resampling algorithm need only recover the true error distribution to provide valid inference under the global null.

Various other resampling approaches that may appear valid in fact violate the assumption. For example, we could fix the design matrix X but resample with replacement the outcome vectors, $\left(Y_{n^{\prime }1},\cdots ,Y_{n^{\prime }W}\right)$, rather than the residuals. Although this approach indeed enforces the global null and preserves the correlation between the outcomes, it fails to preserve the correlations between the outcomes and the adjusted covariates and thus does not recover the distribution of $T^{0}$.

A second incorrect alternative would be to bootstrap parametrically from Equation (1) while enforcing the global null by constraining each $\beta_{w}^{\left(j\right)}:=0$. However, this sequential algorithm fails to entirely preserve the correlations among the outcomes if there are unmeasured variables, beyond the adjusted covariates, that contribute to these correlations. In turn, the distribution of $T^{0}$ is not recovered. A final incorrect alternative would be a generic bootstrap hypothesis test performed by resampling with replacement entire rows of data and then centering the test statistics as in Equation (2). However, this algorithm incorrectly treats the design matrix as random rather than fixed, which would be appropriate for correlation models but not the intended regression models [18]. Additionally, this algorithm can produce data violating the assumptions of standard OLS inference, even when the original data fulfill the assumptions. Suppose, for example, that the design matrix contains only an intercept and a binary exposure of interest, $X_{2}\in \{0,1\}$, and that, for some outcome $Y_{w^{*}}$, we have $\beta_{w^{*}}\neq 0$ (i.e., the alternative hypothesis holds). Then, of course, ${\hat{\epsilon }}_{nw^{*}}$ may be normal, allowing valid OLS inference, despite that $Y_{w^{*}}$ itself may be bimodal with peaks at $E\left[Y_{w^{*}}|X_{2}=0\right]$ and $E\left[Y_{w^{*}}|X_{2}=1\right]$. This generic resampling algorithm retains the bimodality of $Y_{w^{*}}$ while breaking the association between $Y_{w^{*}}$ and $X_{1}$; thus, the resampled residuals ${\hat{\epsilon }}_{nw^{*}}^{\left(j\right)}$ will be bimodal rather than normal [5], and standard inference may fail.

## 4. Generalized setting and notation

To establish theoretical results that hold generally for models other than OLS, we now formalize a more general multiple-testing framework. Suppose that K random variables are measured on N subjects, with the resulting matrix denoted $Z\in {\mathbb{R}}^{N\times K}$. Let $Z_{nk}$ denote, for the $n^{th}$ subject, the $k^{th}$ random variable. Consider a resampling algorithm that generates, for iterate j, a dataset $Z^{\left(j\right)}\in {\mathbb{R}}^{N\times K}$ containing the random vector $\left(Z_{n1}^{\left(j\right)},\cdots ,Z_{nK}^{\left(j\right)}\right)$ for each subject n. There are a total of B resampled datasets. We use the superscript ^“(*j*)”^ to denote random variables, distributions, and statistics in resampled dataset j. Further suppose that we conduct W-tests of point null hypotheses, each at level α. Denote the $w^{th}$ null hypothesis as $H_{0w}$. Let $c_{w,\alpha }$ be the critical value for the test statistic, $T_{w}$, of the $w^{th}$ test. The W-vector of test statistics is $T=\left(T_{1},\cdots ,T_{w}\right)$. We define the “global null” as the case in which all W null hypotheses hold and use the superscript “^0^” generally to denote distributions, data, or statistics generated under the global null.

We treat the observed data, Z, as fixed and conditioned throughout but suppress explicit conditioning for brevity. Define the statistic corresponding to the observed number of α-level rejections as $\hat{\theta }=\sum_{w=1}^{W}1\left\{T_{w}>c_{w,\alpha }\right\}$. Its counterpart in a sample generated under the global null is ${\hat{\theta }}^{0}$ and in a resample j is ${\hat{\theta }}^{\left(j\right)}$. Using F to denote a cumulative distribution function (CDF) and r a non-negative integer at which it is evaluated, we respectively define the true CDF of the number of rejections under the global null, its counterpart in the resamples, and its empirical estimator in the B resamples as:

$$F_{{\hat{\theta }}^{0}}(r)=P\left({\hat{\theta }}^{0}\leq r\right)\;F_{{\hat{\theta }}^{\left(j\right)}}(r)=P\left({\hat{\theta }}^{\left(j\right)}\leq r\right)\;{\hat{F}}_{{\hat{\theta }}^{\left(j\right)}}(r)=\frac{1}{B}\sum_{j^{*}=1}^{B}1\left\{{\hat{\theta }}^{\left(j^{*}\right)}\leq r\right\}$$

We denote almost sure convergence, convergence in probability, convergence in distribution, and ordinary limits respectively as $\text{“}\overset{A.S.}{\underset{N\to \infty }{\to }}\text{”}$, $\text{“}\overset{P}{\underset{N\to \infty }{\to }}\text{”}$, $\text{“}\overset{D}{\underset{N\to \infty }{\to }}\text{”}$ and $\text{“}\vec{N\to \infty }\text{”}$.

## 5. Main results

We now develop theory allowing us to approximate the sampling distribution of $F_{{\hat{\theta }}^{0}}$ through resampling. Specifically, we show that under a certain class of resampling algorithms defined below, the empirical sampling distribution of the number of rejections in the resamples converges to the true distribution of the number of rejections in samples generated under the global null. We chose to characterize the sampling distribution empirically rather than theoretically because it does not appear to have a tractable closed form without imposing assumptions on the correlation structure of the tests and potentially requiring asymptotics on the number of hypothesis tests (Despite the intractable sampling distribution, it is straightforward to derive at least the exact variance of ${\hat{\theta }}^{0}$ if the pairwise correlations between the p-values are known; see Supplementary material). Because simulation error associated with using a finite number of resamples to approximate the CDF of the resampled data can be made arbitrarily small by taking B→∞, we follow convention (e.g., [18]) in ignoring this source of error and considering only asymptotics on N.

### 5.1. An assumption on the resampling algorithm

To establish the main convergence result, we will use the following key assumption stating that, regardless of whether the observed sample was generated under the global null or under an alternative, the resampling algorithm must generate a sampling distribution for $T^{\left(j\right)}$ that converges to the sampling distribution of $T^{0}$ (that is, in samples generated under the global null). We will later discuss resampling algorithms that satisfy this assumption.

#### Assumption 1.

The resampling algorithm used to generate $Z^{\left(j\right)}$ must ensure that $T^{\left(j\right)}\overset{D}{\underset{N\to \infty }{\to }}T^{0}$, or equivalently $F_{T^{\left(j\right)}}\;\vec{N\to \infty }\;F_{T^{0}}$.

Typically, resampling algorithms fulfilling this assumption will need to preserve the correlation structure of all variables in the dataset, except where the global null dictates otherwise. If not, the distribution of the test statistics will usually not be preserved. Additionally, just as the original data are assumed to respect the parametric assumptions of all W hypothesis tests, the resampled data must be generated in a manner that also respects this parametric structure. Otherwise, hypothesis tests conducted on the resampled data may not preserve their nominal α-levels, which again affects the distribution of the test statistics. We show in Supplementary material that Algorithm 1 for OLS models fulfills Assumption 1, as motivated heuristically in Section 3 above.

#### Remark 1.

For Assumption 1 to hold, it is sufficient for T to be a continuous function of Z and for $Z^{\left(j\right)}\;\underset{N\to \infty }{\overset{D}{\to }}\;Z^{0}$. Note that this condition is *not* necessary; for example, [5] propose several algorithms that induce the global null by centering the data themselves by sample estimates, rather than by centering the test statistics as in Algorithm 1 above. In such cases, Assumption 1 may hold without $Z^{\left(j\right)}\underset{N\to \infty }{\overset{D}{\to }}Z^{0}$.

### 5.2. Valid inference on the number of rejections

We now present the main theorem establishing that resampling algorithms fulfilling Assumption 1, such as Algorithm 1 for OLS, also yield valid inference on the number of rejections (see Supplementary material for proof).

#### Theorem 1.

Under Assumption 1, ${\hat{\theta }}^{\left(j\right)}\underset{N\to \infty }{\overset{D}{\to }}{\hat{\theta }}^{0}$.

This theorem implies that valid inference, including the null interval and global test, can be conducted using the distribution of the number of rejections in resamples generated using an algorithm fulfilling Assumption 1.

## 6. Practical use and interpretation

In practice, to estimate the proposed metrics, one would first use a resampling algorithm fulfilling Assumption 1 to generate a large number of resamples under the global null (e.g., B=1,000). Then, the lower and upper bounds of a 95% null interval can be defined as the 2.5^*th*^ and 97.5^*th*^ percentiles of $\left({\hat{\theta }}^{\left(1\right)},\cdots ,{\hat{\theta }}^{\left(j\right)}\right)$, and the p-value for the global test is the empirical tail probability:

$$P_{N}\left({\hat{\theta }}^{\left(j\right)}\geq \hat{\theta }\right)=\frac{1}{B}\sum_{j^{*}=1}^{B}1\left\{{\hat{\theta }}^{\left(j^{*}\right)}\geq \hat{\theta }\right\}$$

We provide an R package, NRejections, to automate the resampling and estimation process for OLS models (see Supplementary material).

The p-value for the global test can be interpreted as the probability of observing at least $\hat{\theta }$ rejections in samples generated under the global null. The null interval can be interpreted as the plausible range of $\hat{\theta }$ in samples generated under the global null. The excess hits, computed as the difference between $\hat{\theta }$ and the upper limit of the null interval, can be interpreted as the number of rejections exceeding what would be expected in 95% of samples under the global null. Note that although the number of excess hits is not necessarily equivalent to the number of false null hypotheses (a point reiterated in Section 9), such an interpretation is in fact valid (under repeated sampling at least 95% of time) for test statistics satisfying subset pivotality, as shown in the next result (see Supplementary material for proof).

### Definition 1 (Subset pivotality).

Define the set of all tested null hypotheses as W. The distribution of $\left(T_{1},\cdots ,T_{W}\right)$ fulfills subset pivotality if, for any K⊆W, the joint distribution of $\left\{T_{w}:w\in K\right\}$ is independent of the truth or falsehood of $\left\{H_{0,w}:w\in W\backslash K\right\}$ [5].

### Theorem 2.

Let $\theta_{hi}$ denote the upper limit of a 95% null interval. Define the set of all tested true null hypotheses as: $K^{\prime }\subseteq W=\left\{w:H_{0,w}\right.\;\text{holds for exactly}\;\left.w\in K^{\prime }\right\}$. Then, under subset pivotality, $\theta_{hi}$ can be interpreted as the maximum number of false positives among the observed rejections at least 95% of the time under repeated sampling, and regardless of the configuration of true and false null hypotheses. That is:

$$\begin{array}{r} P\left(\sum_{w\in K^{\prime }}1\left\{T_{w}>c_{w,\alpha }\right\}>\theta_{hi}|H_{0,w}\;\text{holds for exactly}\;w\in K^{\prime }\right)\\ ≲0.05\; \end{array}$$

where ≲ denotes that the inequality holds asymptotically. Equivalently, 95% of the time under repeated sampling, the number of false null hypotheses is as least the number of excess hits, $\hat{\theta }-\theta_{hi}$.

### Remark 2.

Letting $\alpha_{W}$ denote an arbitrary level of global inference, a general $\left(1-\alpha_{W}\right)\text{\%}$ null interval has an analogous interpretation, again under subset pivotality. That is, its upper limit can be interpreted as the maximum number of false positives at least $\left(1-\alpha_{W}\right)\text{\%}$ of the time under repeated sampling. Equivalently, $\left(1-\alpha_{W}\right)\text{\%}$ of the time under repeated sampling, the number of false null hypotheses is at least the number of excess hits based on a $\left(1-\alpha_{W}\right)\text{\%}$ null interval. These interpretations hold regardless of α, the level of the individual hypothesis tests: for example, a 99% null interval constructed based on individual tests at α=0.05 nevertheless leads to global inference on the number of false positives at the $\alpha_{W}=0.01$ level.

Subset pivotality holds for OLS and a number of other common choices of test statistics [5], in which case the excess hits based on a 95% null interval can thus be interpreted as the minimum number of false null hypotheses such that this statement would hold 95% of the time with repeated sampling. We further illustrate interpretation of the proposed metrics in the following applied example.

## 7. Simulation study

We conducted a simulation study with two objectives. First, we compared the estimated null interval to $\hat{\theta }$ for varying effect sizes in an outcome-wide study and to characterize how its precision depends on the strength of correlation between the hypothesis tests and on the α level used for each test. Second, we assessed the power of global tests conducted using the number of rejections with α=0.05 or α=0.01 for each individual test or derived from the five existing FWER-control methods listed in Table 1. As an additional measure of power, we compared the total number of rejected null hypotheses at a familywise-controlled $\alpha_{W}=0.05$ identified by each method (using the excess hits when considering our method per Theorem 2). All code required to reproduce the simulation study is publicly available (https://osf.io/qj9wa/).

### 7.1. Methods

We generated multivariate standard normal data, comprising 1 covariate (X) and either 40 or 200 outcomes for a fixed N=1,000 subjects. The correlation between each pair of outcomes was $\rho_{YY}$. The correlation between X and a proportion, q, of outcomes was $\rho_{XY}$, with remaining pairs having correlation 0. We manipulated scenario parameters based on the values in Table 2. For scenarios with 40 outcomes, we manipulated these parameters in a full-factorial design. For scenarios with 200 outcomes, we omitted some combinations of parameters such that the true correlation matrix was not positive semidefinite, or such that the empirical correlation matrices were often not positive semidefinite.

Each of 500 simulations per scenario proceeded as follows. We generated an observed dataset according to the scenario. We regressed each outcome $Y_{w}$ on X and conducted a t-test at level α on the coefficient for X. We computed $\hat{\theta }$. For each resampling iterate j (with B=1,000), we resampled per Algorithm 1. We conducted a t-test at level α on the coefficient for X and computed ${\hat{\theta }}^{\left(j\right)}$. We used the quantiles of $\left({\hat{\theta }}^{\left(1\right)},\cdots ,{\hat{\theta }}^{\left(B\right)}\right)$ to construct the null interval, compute the excess hits, and conduct our proposed joint test. We used the t-statistics or p-values from the resamples to conduct joint tests based on the existing methods. For FWER methods, we computed the number of multiplicity-corrected rejections to indicate the number of rejected null hypotheses at familywise $\alpha_{W}=0.05$. For our methods, per Theorem 2, we instead set the number of rejected null hypotheses equal to the maximum of 0 and the excess hits based on a 95% null interval.

We resampled per Algorithm 1 for all resampling-based methods. However, Section 4.2.2 of [5] suggests a different residual-resampling algorithm for OLS in which the resampled residuals alone are used as the resampled outcomes, such that $Y_{nw}^{\left(j\right)}:={\hat{Y}}_{n^{\prime }w}-Y_{n^{\prime }w}$, where $n^{\prime }$ is a resampled observation. Thus, the global null is already enforced in the resampled data, and the test statistics do not require centering. Because the truth or falsehood of each null hypothesis changes the sampling distribution of the OLS coefficient estimates only by a location shift and the subset pivotality assumption described in Section 2 holds for OLS [5, Section 4.2.2], the difference between this algorithm and the one we used is immaterial, as confirmed by additional simulations that are not shown.

### 7.2. Results

We focus primarily on results for scenarios with 40 outcomes because, as noted in the Introduction, correlated hypothesis tests can be particularly problematic in traditional low-dimensional settings. We secondarily discuss results for scenarios with 200 outcomes, in which the relative advantages of our proposed methods generally became more pronounced.

**FIGURE 1:**
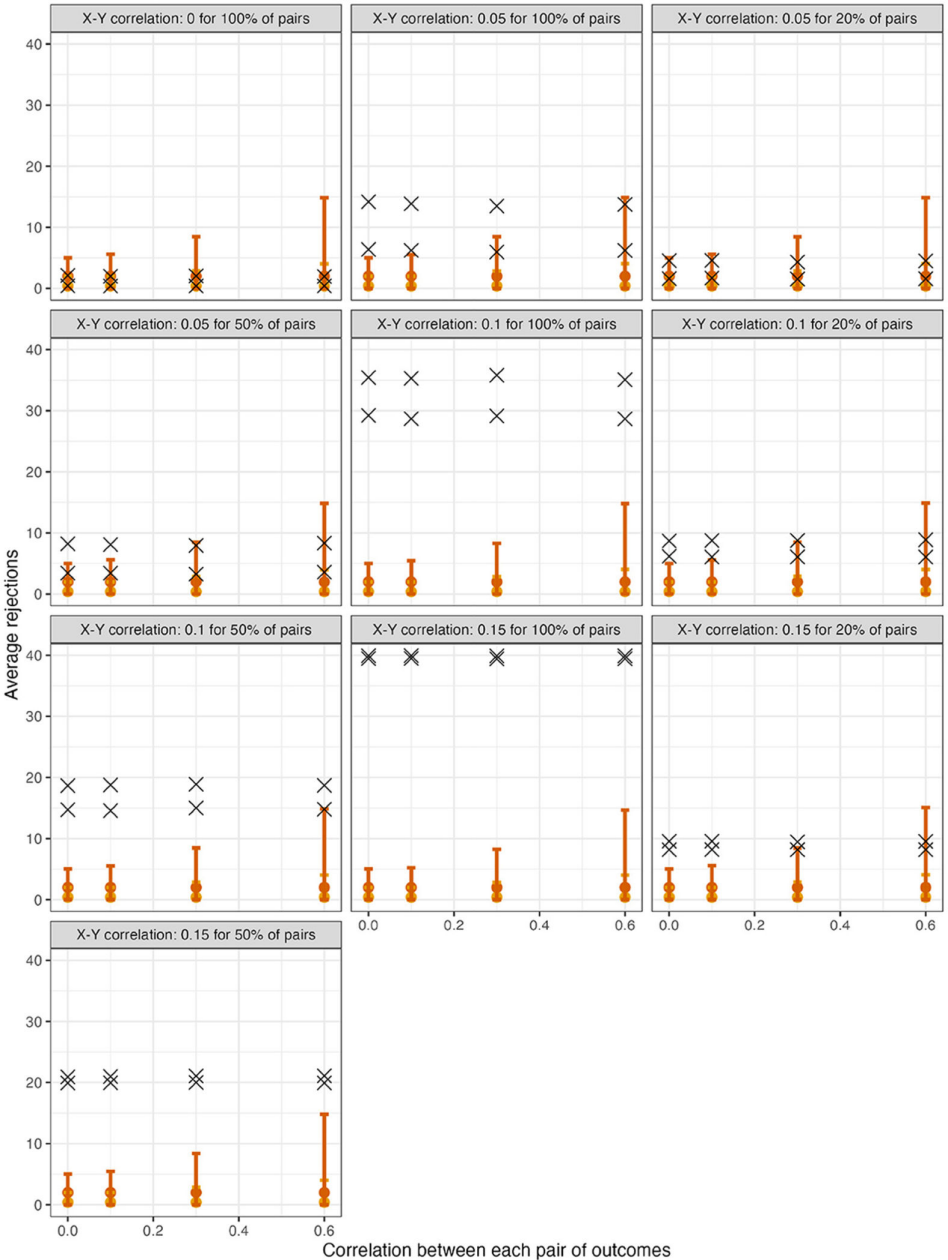
For scenarios with 40 outcomes, 95% null intervals vs. mean rejections in observed datasets (×). Panels: Null and alternative data-generating mechanisms of original samples. Points and error bars: Mean ${\hat{\theta }}^{\left(j\right)}$ and mean limits of null intervals with tests at α=0.01 (yellow) or at α=0.05 (red).

**FIGURE 2:**
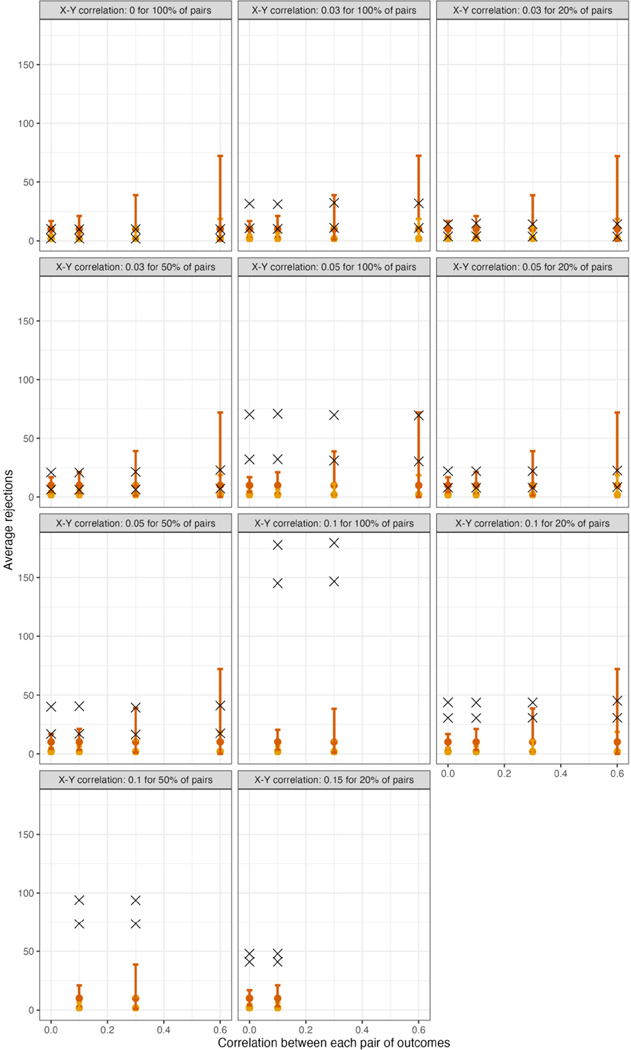
For scenarios with 200 outcomes, 95% null intervals vs. mean rejections in observed datasets (×). Panels: Null and alternative data-generating mechanisms of original samples. Points and error bars: Mean ${\hat{\theta }}^{\left(j\right)}$ and mean limits of null intervals with tests at α=0.01 (yellow) or at α=0.05 (red).

For scenarios with 40 outcomes, Figure 1 displays $\hat{\theta }$ in samples generated under the global null (row 4, panel 1) or under varying alternatives, as well as mean limits of 95% null intervals (For simplicity, Figure 1 does not show all scenarios, but rather excludes some smaller effect sizes. For comprehensive results, see Supplementary material). As expected for a resampling algorithm fulfilling Assumption 1, the null intervals appeared identical regardless of whether the data were generated under the global null. As the pairwise correlation strength between outcomes increased, the null intervals became substantially less precise. For example, with tests conducted at α=0.05, the mean upper limit of the null interval was nearly three times as high for $\rho_{YY}=0.60$ vs. $\rho_{YY}=0$ (i.e., 14.9 vs. 5.0 rejections; see the leftmost and rightmost null intervals within each panel). Thus, with a true effect size of $\rho_{XY}=0.05$ for all pairs (Figure 1, row 1, panel 3), the mean number of observed rejections at α=0.05 (i.e., 13.8) would be within the 95% null interval if the outcomes had correlation strength of $\rho_{YY}=0.60$ (excess hits = 13.8 − 14.9 = −0.8), but would be well outside the null interval, and thus provide stronger evidence for global association, if the outcomes were independent (excess hits = 13.8 − 5.0 = 8.8). For scenarios with 200 outcomes, the corresponding results appear in Figure 2.

**FIGURE 3:**
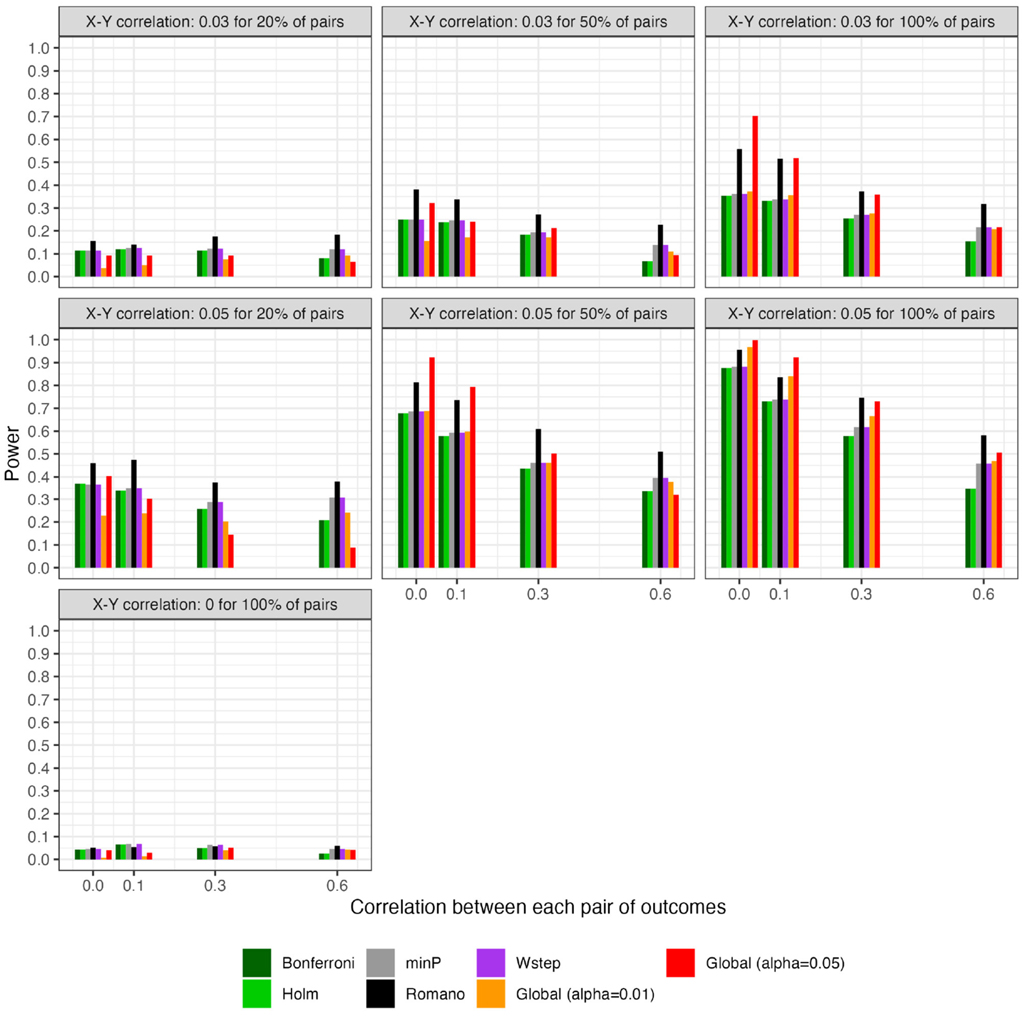
For scenarios with 40 outcomes, power or Type I error of global tests based on existing FWER-control procedures and on the number of rejections. “Global (alpha = 0.01)” and “Global (alpha = 0.05)”: proposed methods. The final panel represents Type I error under the global null.

**FIGURE 4:**
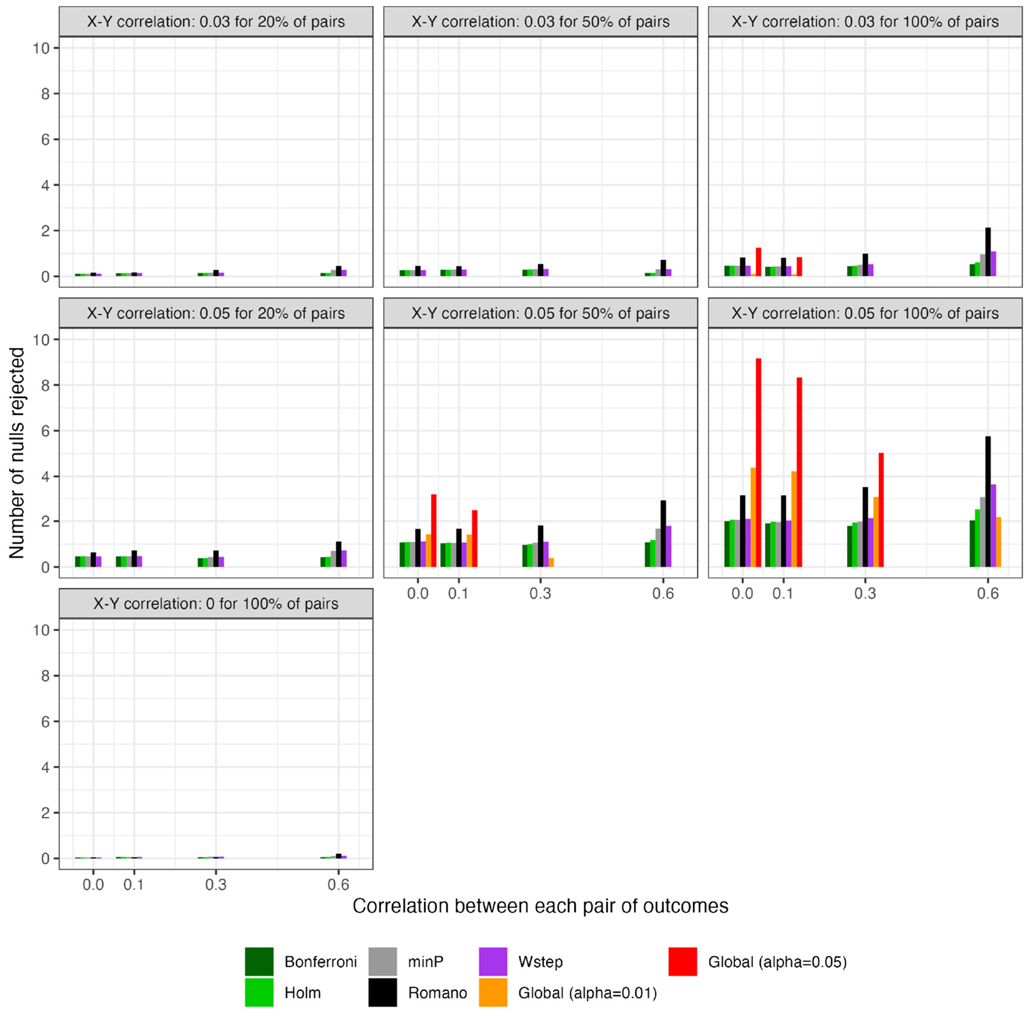
For scenarios with 40 outcomes, number of rejected null hypotheses at familywise-controlled $\alpha_{W}=0.05$ based on existing FWER-control procedures and on the excess hits. “Global (alpha = 0.01)” and “Global (alpha = 0.05)”: proposed methods. Red dashed line: Actual number of false null hypotheses (q×W).

**FIGURE 5:**
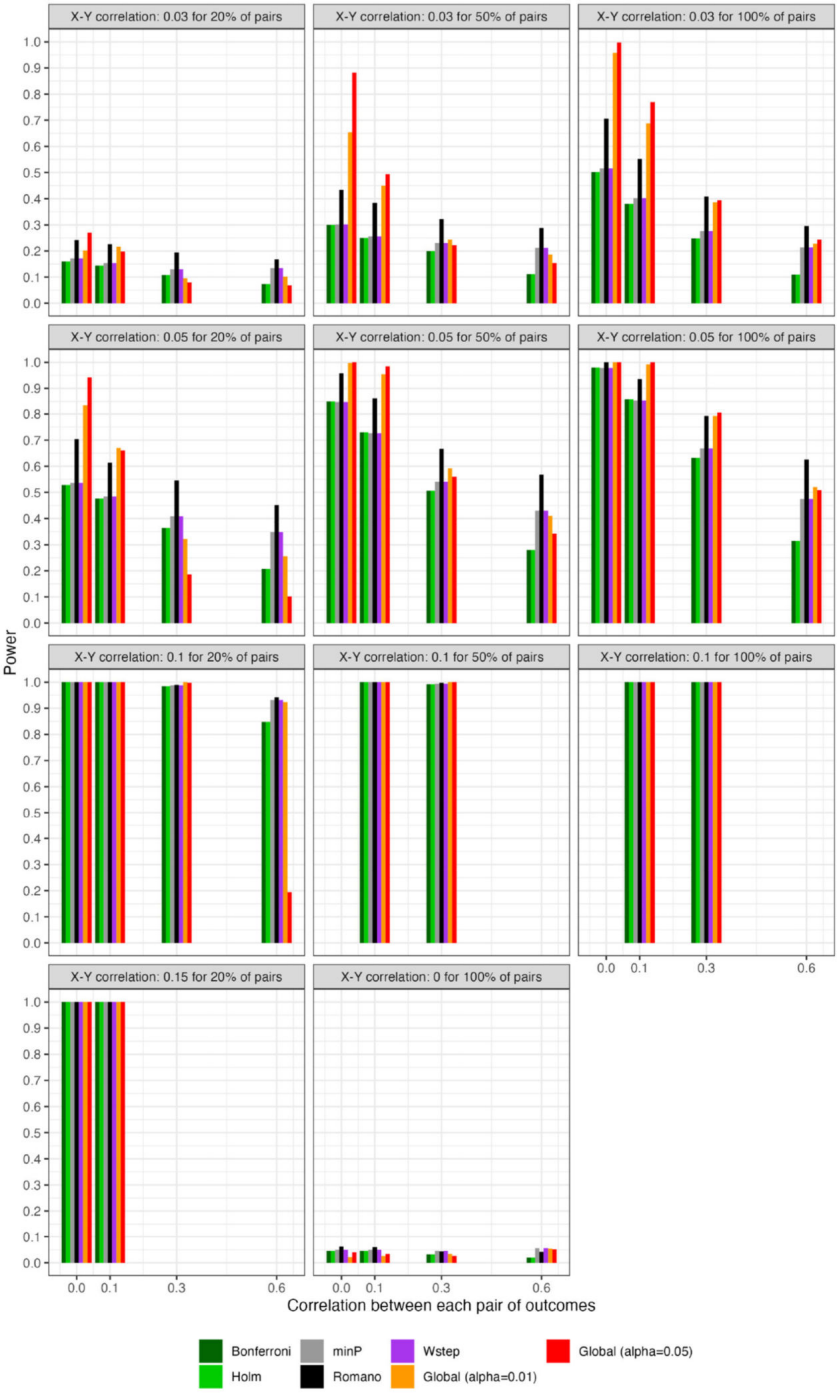
For scenarios with 200 outcomes, power of global tests based on existing FWER-control procedures and on the number of rejections. “Global (alpha = 0.01)” and “Global (alpha = 0.05)”: proposed methods. The final panel represents Type I error under the global null.

**FIGURE 6:**
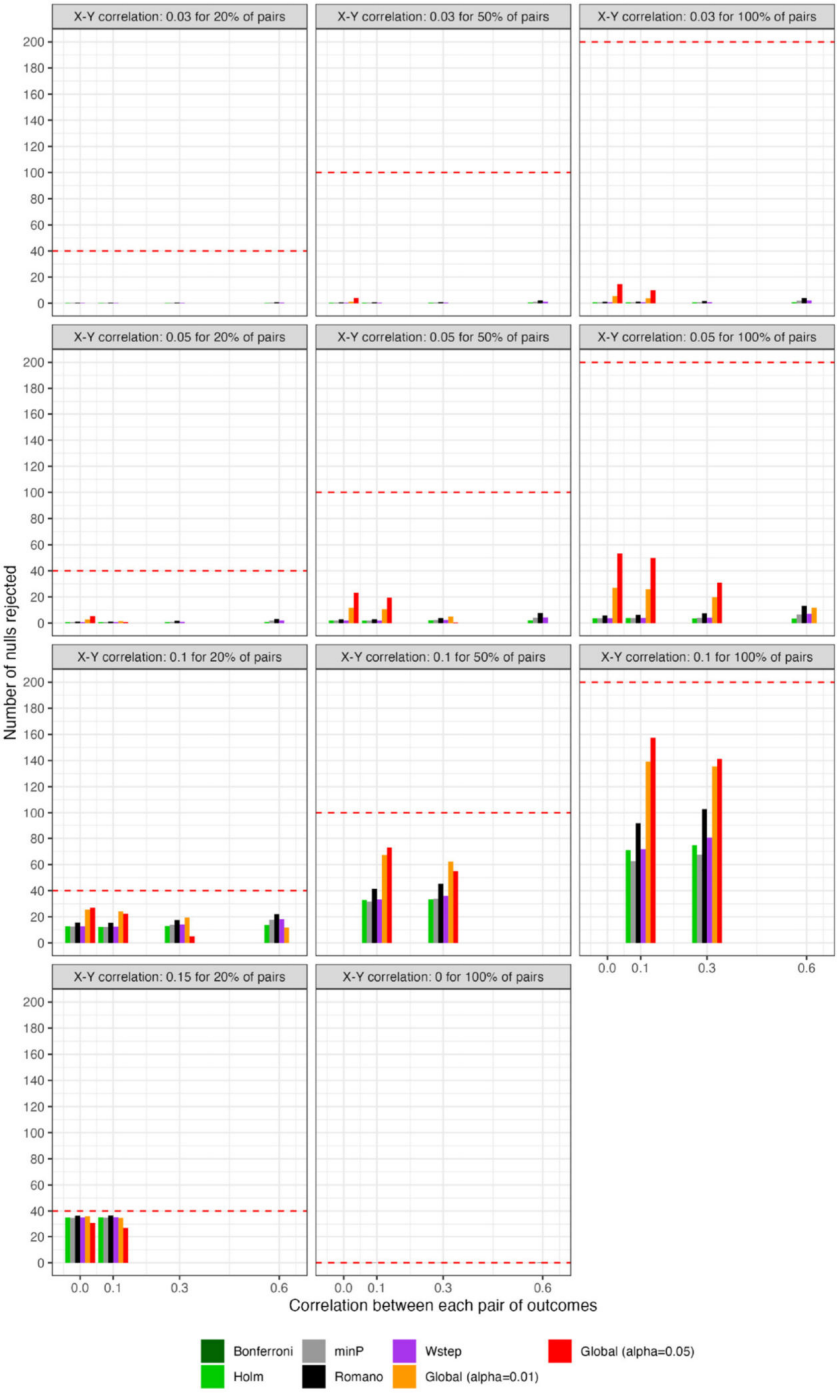
For scenarios with 200 outcomes, number of rejected null hypotheses at familywise-controlled $\alpha_{W}=0.05$ based on existing FWER-control procedures and on the excess hits. “Global (alpha = 0.01)” and “Global (alpha = 0.05)”: proposed methods. Red dashed line: Actual number of false null hypotheses (q×W).

For scenarios with 40 outcomes, Figures 3 and 4, respectively, show the power (or Type I error) of each global test and the number of rejected null hypotheses (Again, we show a subset of scenarios, excluding those in which all methods had nearly 100% power and excluding some intermediate correlation strengths. These scenarios differ from those in Figure 1. Comprehensive results appear in the Supplementary material). As expected, when data were generated under the global null, all methods had approximately nominal or conservative Type I error rates (Figure 3). Our proposed global test achieved its best performance with weakly correlated or independent statistics (Figure 3) and when a moderate to high proportion of alternative hypotheses were true (q>0.20). In such scenarios, our proposed methods sometimes rejected as many as 6.0 more null hypotheses on average than the next-best method (Figure 4). Heuristically, these settings are those in which evidence is diffuse across the multiple tests. Because FWER methods focus on individually adjusting each p-value, each individual hypothesis may have a very low probability of being rejected when evidence is diffuse across tests, such that the resulting global tests have low power. However, the *total* number of rejections may still be considerably larger than the expected number, giving our proposed global test an advantage for power. With 200 outcomes, the relative advantages of our proposed methods became considerably more pronounced (Figures 5, 6). This is because FWER impose increasingly severe penalties on each test as the number of tests increases, whereas our propose consider evidence strength holistically across tests.

In contrast, the power of our methods declined when few alternative hypotheses were true (e.g., q=0.20; Figure 3), likely because in these scenarios, $\hat{\theta }$ would often have been near its expectation under the global null. Simultaneously, the small number of p-values corresponding to the true alternative hypotheses may often have been quite small, improving the power of tests derived from FWER methods. Interestingly, in all scenarios we considered, Romano and Wolf [6]’s method uniformly outperformed methods other than the one we propose; it also outperformed ours with highly correlated test statistics, but not always with weakly correlated or independent statistics. Heuristically, when few alternative hypotheses are true or the test statistics are highly correlated across outcomes, evidence is concentrated to a limited number of tests, rather than diffuse across them. In such settings, our proposed global test may have reduced power compared to FWER methods because there is an inherent loss of information when dichotomizing p-values at α to compute the number of rejections. We return to this point in Section 9 With 200 outcomes, our methods often—though not always—outperformed FWER even in these scenarios (Supplementary material).

Besides [6]’s method, the other existing methods, even the conservative naïve methods, performed comparably to one another (within approximately 10 percentage points of power of one another for nearly all scenarios). Based on simple additional simulations (Supplementary Figure 4), we speculate that this somewhat counterintuitive finding arises because the methods appear to differ primarily in their degree of adjustment for those p-values that are ≫0.05, with the resampling-based methods typically yielding substantially smaller, but still “non-significant”, adjusted values for these large p-values. In contrast, p-values near the 0.05 threshold—those that could potentially affect results of the global test—appear to receive only small and comparable adjustments across all methods. Thus, we speculate that it is rather unlikely that a sample would have all adjusted p-values above 0.05 under a naïve approach, but would have at least one p-value adjusted to below 0.05 under a resampling approach.

## 8. Applied example

Existing epidemiologic analyses have investigated the extent to which an individual’s experience of parental warmth during childhood is associated with the individual’s later “flourishing” in mid-life [20]. Flourishing has been broadly conceived as a state of positive mental health comprising high emotional, psychological, and social wellbeing [21], and reductive analyses that individually assess its theorized components, such as perceived purpose in life and positive affect, may not fully capture potential impacts of the overall experience of flourishing [1, 21].

### 8.1. Methods

Similarly to [20], we conducted longitudinal analyses of a subset of N=2, 697 subjects from the “Mid-life in the United States” (MIDUS) cohort study [22] of 7, 108 adults. In an initial wave of data collection (1995–1996), subjects recalled the parental warmth that they experienced during childhood as an average of separate scales of maternal and paternal warmth. In a second wave (2004–2006), the same subjects reported 13 continuous subscales of flourishing in emotional, psychological, and social domains [21].

We first reproduced [20]’s main analysis by assessing the association between a one-unit increase in standardized parental warmth (i.e., an increase of one standard deviation on the raw scale) with a standardized, continuous composite measure of flourishing (“overall flourishing”), which aggregated the 13 subscales. We conducted similar analyses for the remaining 16 continuous outcome variables in [20]’s analyses, namely the 3 standardized composite scores for each domain (emotional, psychological, and social) treated separately and the 13 individual subscales. All of our analyses controlled for age, sex, race, nativity status, parents’ nativity status, number of siblings, and other childhood family factors. We expected the resulting 17 test statistics to be correlated because of conceptual similarities between the subscale variables and because of the composite measures’ arithmetic relationships with their subscales. Last, to characterize overall evidence strength across the 17 outcomes, we resampled per Algorithm 1 with B=5,000 to estimate the null interval and excess hits (with each test conducted ateither α=0.05 or α=0.01) and to conduct the global test using the number of rejections in individual tests conducted at α=0.05. All data and code required to reproduce these analyses is publicly available and documented (https://osf.io/qj9wa/).

### 8.2. Results

Supplementary Table 1 displays demographic characteristics in our sample. The 17 outcome measures had a median correlation magnitude of |r|=0.39 (minimum = 0.12; maximum = 0.89; 25^*th*^ percentile = 0.28; 75^*th*^ percentile = 0.55). The composite analysis estimated that, controlling for demographics and childhood family factors, individuals reporting an additional standard deviation (SD) of parental warmth in childhood experienced greater mid-life flourishing by, on average, b=0.22 (95% CI: [0.18, 0.26]) SDs.

**FIGURE 7:**
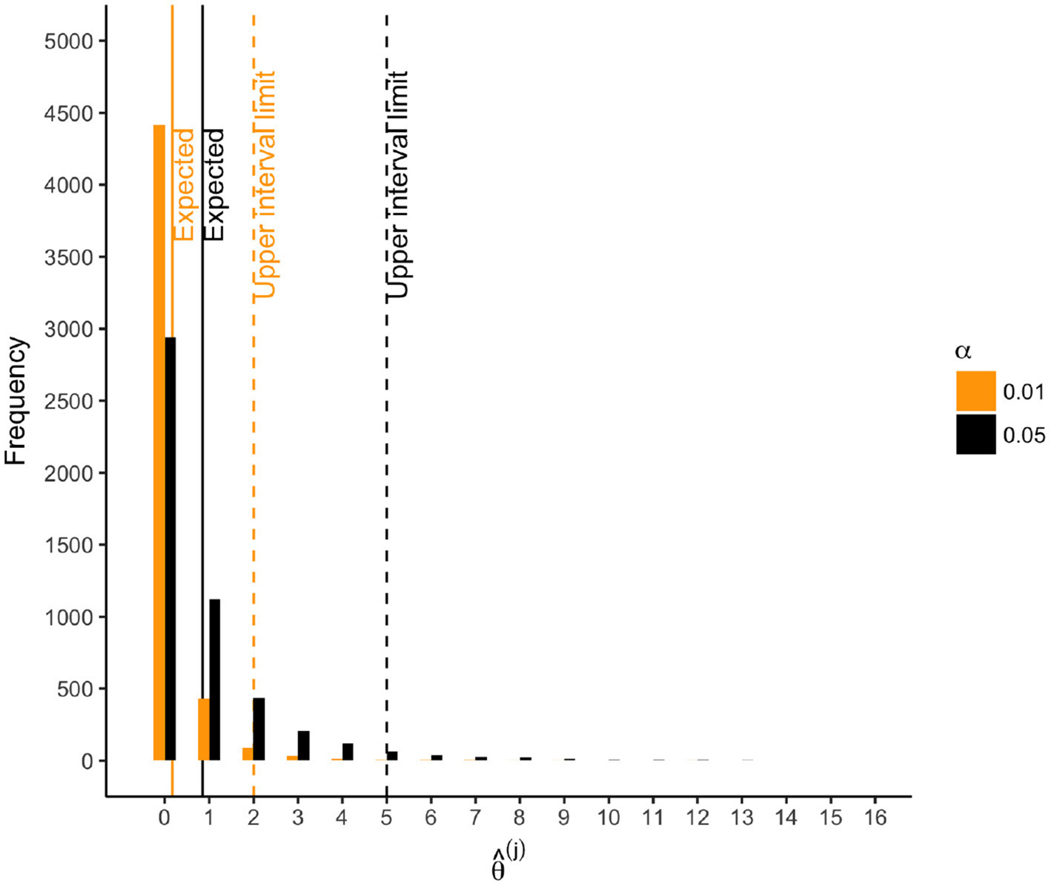
For scenarios with 40 outcomes, number of rejections $\left({\hat{\theta }}^{\left(j\right)}\right)$ for each of 5, 000 resamples. Solid lines: $E\left[{\hat{\theta }}^{0}\right]=\alpha \times 17$. Dashed lines: upper limit of 95% null interval.

Of the 17 outcomes considered individually, all were “significantly” associated with parental warmth at α=0.05 (i.e., $\hat{\theta }$ = 17), and 15 were “significantly” associated at α=0.01. The mean standardized effect size was b=0.14. The directions of all effects suggested that increased parental warmth was associated with improved flourishing outcomes (Table 3). In contrast, if parental warmth were in fact unassociated with any of the outcomes, we would expect 17 × 0.05 = 0.85 rejections with a null interval of [0, 5] at α=0.05 (Figure 7). At α=0.01, we would expect 0.17 rejections with null interval [0, 2] at α=0.01. Thus, at α=0.05 and α=0.01 respectively, we observed 17 − 5 = 12 and 15 − 2 = 13 excess hits above what would be expected in 95% of samples under the global null. Thus, 95% of the time under repeated sampling, at least 13 of the 17 null hypotheses would be false, indicating non-zero associations of parental warmth. Indeed, a global test based on the number of rejections at α=0.05 suggested very strong evidence against the global null (p=0 because every resampled dataset had < 17 rejections; Figure 1). (By comparison, simple inference based on the exact binomial distribution, assuming anticonservatively that the outcomes are independent, yields a too-narrow null interval at α=0.05 of [0, 3] and a global p-value of 0.05^17^ = 7×10^−23^.) Overall, our composite analyses strongly support small effects of parental warmth on composite flourishing, as reported by [20] (Table 3, first row); our novel analyses of $\hat{\theta }$ additionally provide compelling global evidence for associations of parental warmth with flourishing across the 17 outcomes, accounting for their correlation structure.

## 9. Discussion

This paper has characterized global evidence strength across arbitrarily correlated hypothesis tests without being restricted to the setting of high-dimensional analyses. Specifically, we proposed metrics that compare the observed number of test rejections, $\hat{\theta }$, to its expected sampling distribution under the global null. $\hat{\theta }$ is a simple summary measure that seems of natural interest; the proposed metrics help to rigorously ground intuition regarding its behavior when tests are correlated. First, we proposed reporting a null interval for the number of α-level rejections expected in 95% of samples generated under the global null along with the number of excess hits observed above the upper interval limit. Second, we proposed reporting a one-sided test of the global null whose p-value represents the probability of observing at least $\hat{\theta }$ rejections in samples generated under the global null. For OLS models, these metrics can be easily estimated via resampling using our R package, NRejections.

Existing methods that control FWER for arbitrarily correlated tests can also be used to conduct such a global test, so we conducted a simulation study assessing their relative power. To our knowledge, this is the first direct comparison of these methods as global tests, rather than as FWER-control procedures. All methods showed nominal or conservative Type I error rates, as expected theoretically. Our method performed well when tests were independent or weakly correlated and when a moderate to high proportion of alternative hypotheses were true; therefore, it may be most suitable for studies in which the uncorrected p-values are relatively similar to one another, rather than for studies in which a small number of uncorrected p-values are much smaller than the others. The global test based on [6]’s method performed very well overall and, in the OLS scenarios we considered, appeared to uniformly outperform existing methods other than sometimes our own, concerning which [6]’s method was often more powerful, though ours sometimes performed better with weakly correlated or independent tests. In these contexts, considering the excess hits sometimes allowed rejection of many more null hypotheses with familywise control than the next-best method (e.g., an average of 9.17 vs. 3.17 in one such scenario).

Our simulations indicated [6]’s method sometimes has superior power to ours as a global test in low-dimensional settings (e.g., with W=40 tests), though our method more frequently has superior power in higher-dimensional settings (e.g., with W=200 tests). We speculate that when [6]’s method does have superior power, despite its additional need to strongly control FWER, this reflects the loss of information inherent in dichotomizing p-values at α to compute the number of rejections. A more powerful global test might be based, for example, on departures of the observed joint ECDF of the p-values, treated as continuous, from their CDF under the global null, as estimated via resampling methods such as those outlined in this paper and in [5]. However, even in contexts in which a global test derived from [6]’s method does provide better power (though this is not always the case), the null interval and excess hits may still be of interest. More broadly, we view $\hat{\theta }$ and the proposed metrics as useful summaries of global evidence strength that do lose some information in the process of summarization. As such, they supplement, rather than replace, reporting individual, continuous p-values with and without standard multiplicity corrections.

Our consideration of existing methods has focused on repurposing those that adjust individual p-values or critical values. Other existing methods, like our proposed metrics, do directly characterize overall evidence strength and merit some discussion. For example, global inference on regression coefficients for different outcomes can be conducted using multivariate regression [23] or [24]’s “seemingly unrelated regressions” generalization. However, these approaches only modestly improve efficiency compared to that achieved in W separate OLS models, and when the design matrix is shared across models, coefficient estimates are identical to those in OLS models [25]. Another approach to global inference is to meta-analyze the effect sizes from each analysis [10]. Compared to direct analysis of the raw data, meta-analysis is likely to be inefficient. Alternatively, one could conduct global inference on a reduced number of outcomes by constructing composite measures (as in the applied example) or applying statistical dimension reduction, such as principal components analysis, though some information is lost. Another class of methods conducts global inference through the direct combination of p-values, though these methods almost always assume independence or a known parametric dependence structure ([26–29]; but see [30] for a non-parametric exception) or apply only for specific types of genetic data [31].

When interpreting our proposed metrics, it is important to note that they characterize the sampling distribution under, specifically, the global null. Thus, rejecting the global test at α=0.05 indicates that there is no more than a 5% probability of observing at least $\hat{\theta }$ rejections in samples generated under the global null. The excess hits can be interpreted as the minimum number of false null hypotheses 95% of the time under repeated sampling only under subset pivotality and otherwise must not be misinterpreted as such. Statements to this effect can be also made using procedures that strongly control FWER. By construction, these procedures ensure that for a familywise $\alpha_{W}=0.05$, in 95% of samples generated under any configuration of null and alternative hypotheses, each rejected test will represent a true positive; therefore, the number of rejections based on inference adjusted to strongly control FWER can also be interpreted as the minimum number of false null hypotheses 95% of the time under repeated sampling. There are also interesting methods designed specifically for providing confidence statements regarding the number of false null hypotheses [32]. Because these methods require resampling under various intersections of the null hypotheses, they appear well-suited to hypothesis tests that span multiple exposures, for which intersection testing can simply involve testing a single regression model on all the exposures against various nested null models. However, these methods appear less suitable to hypothesis tests spanning multiple outcomes, for which intersection testing is more challenging.

Our proposed methods are valid for arbitrary hypothesis tests as long as data are resampled such that the resampled test statistics converge in distribution to the distribution they would have under the global null. To this end, an additional contribution of this paper is the theoretical justification of residual resampling for OLS models in the context of multiple testing, informed by [18]’s work for a single regression model and [5]’s related algorithms. Indeed, a central challenge for resampling-based methods for multiple testing in general is the design of valid resampling procedures. The present theory supports using residual resampling under the global null for OLS in the context of our methods, of FWER control [5–7], of FDP control [17], and of corrections for “data snooping” [33]. We focused on OLS-based hypothesis tests because of their generality and ability to subsume many common tests. However, for certain other tests, such as those based on GLMs with non-identity link functions, validly resampling under the global null appears to be an open problem, although algorithms have been developed outside the multiple testing context for confidence intervals (e.g., [34]) and, under additional assumptions, for permutation hypothesis tests [35]. Other estimators, such as those using propensity score matching, pose challenges for resampling because the estimators lack certain smoothness properties; these challenges arise even without the need to enforce the global null. Algorithms fulfilling Assumption 1 for such estimators could potentially use subsampling to relax some of the smoothness assumptions of with-replacement resampling [36].

Correlated test statistics can naturally arise not only when testing multiple associations between exposures and outcomes, but also when multiple hypothesis tests are used to investigate the same question, as in “data snooping” [33] or “p-hacking” [37]. For example, investigators often fit several regression models to investigate the same association of interest, adjusting for different sets of covariates or using different subsets of the data. When investigators report selectively among these findings, for example by reporting only significant results, this can lead to substantial bias in the published literature [38, 39]. Elsewhere, we gave formal conditions for when p-hacking will lead to bias [40]. Situating these “researcher degrees of freedom” within a formal multiple testing context [33], rather than merely reporting a single result chosen *post hoc*, could help reduce unnecessary false positives in the literature and may additionally foster a more balanced overall view of the evidence. Excessive focus on null-hypothesis testing has itself contributed to selective reporting in the published literature, and we would therefore suggest reporting our proposed metrics along with appropriate measures of effect size [41]. Correlated hypothesis tests can also arise when investigators re-analyze a single dataset using numerous different model specifications as a sensitivity analysis, a “multiverse” approach that has rapidly gained traction in the social sciences [2]. Our proposed metrics provide one approach to summarizing evidence in such settings; for example, the p-value from the global test could help characterize evidence supporting a false null hypothesis in at least one of the multiple model specifications.

In summary, the number of rejections across correlated hypothesis tests can be a useful summary measure of overall evidence strength when reported with metrics such as a null interval, the number of excess hits, and a test of the global null. Reporting these metrics alongside p-values with and without standard multiplicity corrections may provide a richer view of global evidence strength than corrected inference alone.

## Supplementary Material

Supplement

## Figures and Tables

**TABLE 1:** Selected existing methods for strong FWER control with correlated hypothesis tests.

Method	Type	Means of handling correlation
Bonferroni	1-step	Conservatively making no assumptions
Holm	Step-down	Conservatively making no assumptions
minP	1-step	Resampling under global null to estimate correlation structure
Wstep	Step-down	Same as above
Romano	Step-down	Resampling without restrictions to estimate correlation structure

**TABLE 2:** Possible values of simulation parameters.

$\rho_{XY}$	$\rho_{YY}$	q	α
0.03	0	0	0.01
0.05	0.10	0.20	0.05
0.10	0.30	0.50	
0.15	0.60	1	

**TABLE 3:** OLS estimate $\left(\hat{\beta }\right)$ characterizing association of a 1-SD increase in parental warmth with each of 17 standardized flourishing outcomes, adjusting for all covariates in Supplementary Table 1.

Outcome	$\hat{\beta }$[95% CI]	p-value
**Overall and domain composites**		
Overall flourishing	0.22 [0.18, 0.26]	< 2 × 10^−16^
Emotional wellbeing	0.21 [0.17, 0.25]	< 2 × 10^−16^
Social wellbeing	0.13 [0.08, 0.17]	2 × 10^−9^
Psychological wellbeing	0.20 [0.16, 0.24]	< 2 × 10^−16^
**Emotional wellbeing subscales**		
Positive affect	0.19 [0.15, 0.23]	< 2 × 10^−16^
Life satisfaction	0.19 [0.15, 0.23]	< 2 × 10^−16^
**Social wellbeing subscales**		
Meaningfulness of society	0.04 [0, 0.08]	0.048
Social integration	0.15 [0.11, 0.19]	5 × 10^−13^
Social acceptance	0.09 [0.05, 0.13]	3 × 10^−5^
Social contribution	0.09 [0.05, 0.13]	1 × 10^−5^
Social actualization	0.06 [0.02, 0.11]	0.002
**Psychological wellbeing subscales**		
Autonomy	0.08 [0.04, 0.12]	3 × 10^−4^
Environmental mastery	0.14 [0.09, 0.18]	6 × 10^−11^
Personal growth	0.11 [0.07, 0.15]	4 × 10^−7^
Positive relations	0.25 [0.21, 0.29]	< 2 × 10^−16^
Purpose in life	0.05 [0.01, 0.09]	0.018
Self-acceptance	0.22 [0.18, 0.26]	< 2 × 10^−16^

Inference is not multiplicity-corrected.

## Data Availability

Publicly available datasets were analyzed in this study. This data can be found here: all code required to reproduce the applied example and simulation study is publicly available (https://osf.io/qj9wa/). The dataset used for the applied example is publicly available through the Inter-University Consortium for Political and Social Research (ICPSR); we detail how to access the dataset in a public repository (https://osf.io/u69s2/).
