# Supplementary material for: New metrics for multiple testing with correlated outcomes
Source: Front Appl Math Stat. Author manuscript; Available in PMC 2025 May 29. (PMC12122011; doi:10.3389/fams.2023.1151314)
Supplement: Supplement [file NIHMS2073645-supplement-Supplement.pdf]

# *Supplement:* New Metrics for Multiple Testing with Correlated Outcomes

## Contents


**1 Exact variance under global null** **2**

**2 Justification of Algorithm 1** **2**

**3 Proof of Theorem 1** **9**

**4 Proof of Theorem 2** **10**

**5 Additional results for the applied example** **12**

**6 Additional simulation results** **14**

6.1 Comparison of $p$-values adjusted by existing methods . . . . . . . . . . . . . 17

**7 Introduction to the package `NRejections`** **17**

## 1. Exact variance under global null

Let $p_w^0$ be the $p$-value in the $w^{th}$ test under the global null, treated as a random variable. Then we have:

$$\begin{aligned}
\mathrm{Var}\left(\widehat{\theta}^0\right) &= \mathrm{Var}\left(\sum_{w=1}^{W} 1\{p_w^0 < \alpha\}\right) \\
&= \sum_{w=1}^{W} \mathrm{Var}\left(1\{p_w^0 < \alpha\}\right) + 2 \sum_{1 \le i < j \le W} \mathrm{Cov}\left(1\{p_i^0 < \alpha\}, 1\{p_j^0 < \alpha\}\right) \\
&= W\alpha\left(1-\alpha\right) + 2 \sum_{1 \le i < j \le W} E\left[1\{p_i^0 < \alpha, p_j^0 < \alpha\}\right] \\
&- E\left[1\{p_i^0 < \alpha\}\right] E\left[1\{p_j^0 < \alpha\}\right] \\
&= W\alpha\left(1-\alpha\right) + 2 \sum_{1 \le i < j \le W} \left[P\left(p_i^0 < \alpha, p_j^0 < \alpha\right) - \alpha^2\right]
\end{aligned}$$

## 2. Justification of Algorithm 1

In Theorem 5 below, we show that Algorithm 1 satisfies Assumption 1, as given in the main text. The development of the proof is structured as follows. We make a regularity assumption (Assumption 2) and define how we will metrize convergence of the resampled test statistics (Definition 2). We bound the distance metric for certain types of random vectors (Lemma 1), in turn allowing us to bound the distance between the estimated sampling distribution in the resamples and the true sampling distribution to which the former should converge (Theorem 3). Using the latter bound, a triangle inequality argument, and convergence results regarding each term of the triangle inequality (Lemmas 2 and 3), we show the needed convergence result for the coefficient estimates (Theorem 4) and finally for the test statistics (Theorem 5).

First, assume the following regularity condition on the design matrix, which will later be relevant for the convergence of the coefficient estimates:

**Assumption 2** (Regularity condition)**.** *Suppose without loss of generality that the regression covariate of interest is $X_2$. Correspondingly, let $B \in \mathbb{R}^{N \times 1}$ be the transposed second row of $(X'X)^{-1}X'$, or equivalently the first column of $X(X'X)^{-1}$. (More generally, if the covariate of interest is the $i^{th}$ variable in the design matrix, then $B$ is defined as the $i^{th}$ row or column.) Assume that for some constant $k > 0$:*

$$N \cdot B'B \xrightarrow[N \to \infty]{P} k$$

$$\Leftrightarrow N \sum_{n=1}^{N} [(X'X)^{-1}X']_{2n}^2 \xrightarrow[N\to\infty]{P} k$$

*where* $[(X'X)^{-1}X']_{2n}$ *denotes a matrix entry.*

This assumption holds under the following sufficient conditions. Heuristically, these state that, for each regression model, the asymptotic standard errors of all $p$ regression coefficients are finite (A1), that the covariates have finite expectations and are not completely collinear (A2), and that the regression model does not fit perfectly (A3).

**Proposition 1** (Sufficient conditions)**.** *Let* $\mathcal{I}_{ij}$ *denote an entry of the expected Fisher information matrix for an individual observation in the* $w^{th}$ *regression. Then Assumption 2 holds if, for all* $w$*:*

$$\mathcal{I}_{ii} > 0 \; \forall \; i \in \{1, \cdots, p\} \tag{A1}$$

$$E[X_{ni}X_{nj}] < \infty \; \forall \; i, j \in \{1, \cdots, p\} \tag{A2}$$

$$\sigma_w^2 > 0 \tag{A3}$$

*Proof of Proposition 1.* Let $\widehat{\gamma}_{iw}$ be the $i^{th}$ coefficient estimate in the $w^{th}$ regression, such that $\widehat{\gamma}_{2w} = \widehat{\beta}_w$, the estimate of interest. Thus, let $\widehat{\alpha}_w = [\widehat{\gamma}_{1w}, \widehat{\beta}_w, \widehat{\gamma}_{3w}, \cdots, \widehat{\gamma}_{pw}]'$ be the $p$-vector of estimates in the $w^{th}$ regression. Denote a pairwise covariance $\text{Cov}_{ij} = \text{Cov}\left(\widehat{\gamma}_{iw}, \widehat{\gamma}_{jw}\right)$, and similarly denote a pairwise correlation as $\rho_{ij}$. Then the estimated covariance of $\widehat{\beta}_w$ with $\widehat{\gamma}_{iw}$ is:

$$\begin{aligned} \widehat{\text{Cov}}_{2i} &= \widehat{\rho}_{2i} \cdot \widehat{\text{SE}}\left(\widehat{\beta}_w\right) \cdot \widehat{\text{SE}}\left(\widehat{\gamma}_{iw}\right) \\ &= \widehat{\rho}_{2i} \cdot \frac{1}{\sqrt{N\widehat{\mathcal{I}}_{22}}} \cdot \frac{1}{\sqrt{N\widehat{\mathcal{I}}_{ii}}} \end{aligned} \tag{1}$$

With the left-hand side of Assumption 2 in view, we have:

$$(X'X)^{-1} = \frac{1}{\widehat{\sigma}_w^2} \begin{bmatrix} \widehat{\text{Cov}}_{11} & \cdots & \widehat{\text{Cov}}_{1p} \\ \widehat{\text{Cov}}_{21} & \cdots & \widehat{\text{Cov}}_{2p} \\ \vdots & & \vdots \\ \widehat{\text{Cov}}_{p1} & \cdots & \widehat{\text{Cov}}_{pp} \end{bmatrix}$$

$$[(X'X)^{-1}X']_{2n} = \frac{1}{\widehat{\sigma}_w^2} \left[\widehat{\text{Cov}}_{21} \cdots \widehat{\text{Cov}}_{2p}\right] \left[X_{n1} \cdots X_{np}\right]'$$

$$N \sum_{n=1}^{N} [(X'X)^{-1}X']_{2n}^2 = N \sum_{n=1}^{N} \left( \sum_{i=1}^{p} \frac{1}{\widehat{\sigma}_w^2} \widehat{\text{Cov}}_{2i} X_{ni} \right)^2$$

$$= N \frac{1}{\widehat{\sigma}_w^4} \sum_{n=1}^{N} \left( \sum_{i=1}^{p} \sum_{j=1}^{p} \widehat{\text{Cov}}_{2i} \widehat{\text{Cov}}_{2j} X_{ni} X_{nj} \right)$$

$$= N \frac{1}{\widehat{\sigma}_w^4} \sum_{i=1}^{p} \sum_{j=1}^{p} \widehat{\text{Cov}}_{2i} \widehat{\text{Cov}}_{2j} \sum_{n=1}^{N} X_{ni} X_{nj}$$

Applying Equation (1) yields:

$$= \frac{1}{\widehat{\sigma}_w^4} \sum_{i=1}^{p} \sum_{j=1}^{p} \widehat{\rho}_{2i} \ \widehat{\rho}_{2j} \ \frac{1}{\widehat{\mathcal{I}}_{22} \sqrt{\widehat{\mathcal{I}}_{ii} \widehat{\mathcal{I}}_{jj}}} \cdot \frac{1}{N} \sum_{n=1}^{N} X_{ni} X_{nj}$$

$$\xrightarrow[N \to \infty]{P} \frac{1}{\sigma_w^4} \sum_{i=1}^{p} \sum_{j=1}^{p} \rho_{2i} \ \rho_{2j} \ \frac{1}{\mathcal{I}_{22} \sqrt{\mathcal{I}_{ii} \mathcal{I}_{jj}}} \ E[X_{ni} X_{nj}]$$

If the sufficient conditions (A1)–(A3) above are fulfilled, this is a finite constant, as required. □

We will consider the validity of the bootstrap in terms of convergence on the Mallows-Wasserstein metric, a conventional choice that is defined as follows (DasGupta, 2008; Freedman, 1981).

**Definition 2** (Mallows-Wasserstein metric)**.** *Let $G_A$ and $G_B$ be arbitrary marginal distribution functions for random vectors $A \in \mathbb{R}^W$ and $B \in \mathbb{R}^W$, respectively. Then a form of Mallows-Wasserstein distance between $G_A$ and $G_B$ is the infimum, taken over all possible joint distributions for $(A, B)$ such that $A \sim G_A$ and $B \sim G_B$ marginally, of the expected $L_2$ distance between $A$ and $B$:*

$$d_2 \left( G_A, G_B \right) := \inf_{\substack{A \sim G_A \\ B \sim G_B}} E \left[ ||A - B||^2 \right]^{1/2}$$

We proceed to prove that the residual-resampling bootstrap is consistent with respect to the Mallows-Wasserstein metric in a development roughly following those of Freedman (1981) and Bickel & Freedman (1981), who considered the asymptotic validity of residual resampling in recovering the sampling distribution of a $p$-vector of coefficient estimates from a single multiple linear regression model. Here, we extend this work to consider the sampling distribution of $\widehat{\beta}^W$. We first establish a lemma bounding the Mallows-Wasserstein distance between the distributions of two random vectors constructed as products of different random matrices with a single fixed vector.

**Lemma 1.** *Let $C^*$ and $D^* \in \mathbb{R}^{W \times N}$ be random matrices from a specific joint distribution, and let $B \in \mathbb{R}^{N \times 1}$ be a fixed vector. Let $G_C$ and $G_D$ be the resulting marginal distribution*

*functions of the vectors* $C^*B$ *and* $D^*B \in \mathbb{R}^{W\times 1}$*, respectively. Then:*

$$d_2\left(G_C, G_D\right)^2 \leq tr\Big\{BB' \cdot E\big[(C^* - D^*)'(C^* - D^*)\big]\Big\}$$

*Proof.* First note that $d_2\left(G_C, G_D\right)^2$ is the infimum of the expectation over all possible joint distributions with marginals $G_C$ and $G_D$, whereas the quantity $E\Big[||C^*B - D^*B||^2\Big]$ is the expectation for a particular such joint distribution (i.e., the one giving rise to $C^*B$ and $D^*B$). We therefore have the inequality:

$$\begin{aligned}
d_2\left(G_C, G_D\right)^2 &\leq E\Big[||C^*B - D^*B||^2\Big] \\
&= E\Big[\text{tr}\{\underbrace{(C^*B - D^*B)}_{W\times 1}\underbrace{(C^*B - D^*B)'}_{1\times W}\}\Big] \\
&= E\Big[\text{tr}\{\underbrace{(C^* - D^*)}_{W\times N}\underbrace{BB'}_{N\times N}\underbrace{(C^* - D^*)'}_{N\times W}\}\Big] \\
&= E\Big[\text{tr}\{\underbrace{BB'(C^* - D^*)'(C^* - D^*)}_{N\times N}\}\Big] \\
&= \text{tr}\Big\{E\big[BB'(C^* - D^*)'(C^* - D^*)\big]\Big\} \\
&= \text{tr}\Big\{BB' \cdot E\big[\underbrace{(C^* - D^*)'(C^* - D^*)}_{N\times N}\big]\Big\}
\end{aligned}$$

□

The next theorem bounds the distance between the true sampling distribution of the estimated coefficients and the estimated sampling distribution in the resamples in terms of the distance between the sampling distribution of the true errors and the resampled residuals.

**Theorem 3.** *Let* $F$ *denote the distribution function of the true errors for the* $W$ *regression models,* $(\epsilon_{n1}, \cdots, \epsilon_{nW})$*, and let* $\widehat{F}_N$ *denote the empirical distribution function of the residuals, which is used to approximate* $F$ *in Algorithm 1. Let* $\Psi(F)$ *denote the distribution of the standardized coefficient estimates,* $\sqrt{N}\left(\widehat{\beta}^W - \beta^W\right)$*, that are constructed as a function of the true error distribution;* $\Psi(F)$ *therefore represents the true sampling distribution to which a valid bootstrapped sampling distribution must converge. In contrast, let* $\Psi(\widehat{F}_N)$ *be the distribution of the standardized coefficient estimates in the resamples,* $\sqrt{N}\left(\widehat{\beta}^{W(j)} - \widehat{\beta}^W\right)$*, in which the empirical distribution of the residuals is used to approximate the true distribution.*

*As in Assumption 2, let* $B \in \mathbb{R}^{N\times 1}$ *be the transposed second row of* $(X'X)^{-1}X'$*. Then:*

$$d_2\left(\Psi(F), \Psi(\widehat{F}_N)\right)^2 \leq N \cdot tr\{BB'\} \cdot d_2\left(F, \widehat{F}_N\right)^2$$

*Proof.* Let $U_w' \in \mathbb{R}^{1\times N} = [U_{1w}, \cdots, U_{Nw}]$ such that $(U_{n1}, \cdots, U_{nW}) \sim F$ and:

$$C \in \mathbb{R}^{W\times N} = \begin{bmatrix} — & U_1' & — \\ & \vdots & \\ — & U_W' & — \end{bmatrix} = \begin{bmatrix} U_{11} & \dots & U_{N1} \\ U_{12} & \dots & U_{N2} \\ \vdots & & \vdots \\ U_{1W} & \dots & U_{NW} \end{bmatrix}$$

In general for multiple regression, we have $\widehat{\beta} - \beta = (X'X)^{-1} X'\epsilon$. Thus, we can express $\Psi(F)$ as the distribution of the $W$-vector:

$$\sqrt{N}\left(\widehat{\beta}^W - \beta^W\right) = \sqrt{N}\begin{bmatrix} \left[(X'X)^{-1}X'U_1\right]_2 \\ \vdots \\ \left[(X'X)^{-1}X'U_W\right]_2 \end{bmatrix} = \sqrt{N}\begin{bmatrix} U_1'B \\ \vdots \\ U_W'B \end{bmatrix} = \sqrt{N}\cdot CB$$

whose $w^{th}$ element pertains to the regression coefficient for $X_2$ in the $w^{th}$ regression. Let $D$ be the counterpart of $C$ with $\left(\widehat{U}_{n1}, \cdots, \widehat{U}_{nW}\right) \sim \widehat{F}_N$ in place of $(U_{n1}, \cdots, U_{nW})$.

In view of Lemma 1, note that the entries of the matrix $(C-D)'(C-D) \in \mathbb{R}^{N\times N}$ are:

$$\begin{aligned} [(C-D)'(C-D)]_{kj} &= \sum_{w=1}^{W} [(C-D)']_{kw}[C-D]_{wj} \\ &= \sum_{w=1}^{W} [C-D]_{wk}[C-D]_{wj} \\ &= \sum_{w=1}^{W} \left(U_{kw} - \widehat{U}_{kw}\right)\left(U_{jw} - \widehat{U}_{jw}\right) \end{aligned}$$

We have $E\Big[\left(U_{kw} - \widehat{U}_{kw}\right)\left(U_{jw} - \widehat{U}_{jw}\right)\Big] = \text{Cov}\left(U_{kw} - \widehat{U}_{kw}, U_{jw} - \widehat{U}_{jw}\right)$, but for all $k \neq j$, the covariance is 0 because the observations are independent. Thus, letting $I^N$ denote the $N \times N$ identity matrix, we have that $E[(C-D)'(C-D)]$ is a diagonal matrix such that:

$$E[(C-D)'(C-D)] = I^N \cdot E\Big[\sum_{w=1}^{W}\left(U_{jw} - \widehat{U}_{jw}\right)^2\Big] \tag{2}$$

which holds for any observation $j$ because they are identically distributed. In order to apply Lemma 1, we now restrict attention to a special choice of $C$ and $D$. First note that, by definition:

$$d_2\left(F, \widehat{F}_N\right)^2 = \inf_{\substack{\left(U_{j1},\cdots,U_{jW}\right)\sim F \\ \left(\widehat{U}_{j1},\cdots,\widehat{U}_{jW}\right)\sim \widehat{F}_N}} E\Big[\sum_{w=1}^{W}\left(U_{jw} - \widehat{U}_{jw}\right)^2\Big] \tag{3}$$

Now let $C^* \in \mathbb{R}^{W \times N}$ and $D^* \in \mathbb{R}^{W \times N}$ be a pair of random matrices constructed using random vectors $(U_{j1}, \cdots, U_{jW})$ and $\left(\widehat{U}_{j1}, \cdots, \widehat{U}_{jW}\right)$ that follow the infimum-attaining joint distribution in Equation (3); that is, such that:

$$E[(C^* - D^*)' (C^* - D^*)] = I^N \cdot d_2 \left(F, \widehat{F}_N\right)^2$$

per the representations in Equations (2) and (3). (Such a choice exists by Bickel & Freedman (1981)'s Lemma 8.1.) The result then follows immediately from applying Lemma 1, setting $G_C = \Psi(F)$, $G_D = \Psi\left(\widehat{F}_N\right)$, and $B, C^*$, and $D^*$ as defined above and pulling the scalar $\sqrt{N}$ outside the squared distance. □

Next, to apply the bound in Theorem 3, we will first bound the term on the right-hand side using a triangle inequality, which applies because $d_2(\cdot, \cdot)$ is a metric (Bickel & Freedman, 1981). To this end, let $F_N$ denote the unobserved empirical distribution function of the true error vector, $\epsilon^W$. Then we have the following triangle inequality:

$$d_2\left(\widehat{F}_N, F\right) \leq d_2\left(\widehat{F}_N, F_N\right) + d_2\left(F_N, F\right) \tag{4}$$

The first term on the right-hand side relates the empirical distribution of the residuals to the empirical distribution of the true errors (which are both discrete distributions taking $N$ values); the second term relates the latter empirical distribution to the true error distribution (which is continuous). The next two lemmas bound the terms on the right-hand side of Equation (4); we will later use them to bound the left-hand side.

**Lemma 2.** *For the expectation of the first term on the right-hand side of Equation (4):*

$$E\Big[d_2\left(\widehat{F}_N, F_N\right)\Big] \xrightarrow[N \to \infty]{} 0$$

*Proof.* As in Definition 2, let $U \sim \widehat{F}_N$ and $V \sim F_N$ be arbitrary random variables in $\mathbb{R}^W$ that follow the empirical marginal distributions of the residuals and of the true errors. Denote their elements $(U_1, \cdots, U_W)$ and $(V_1, \cdots, V_W)$. Let $(U^*, V^*)$ be the special choice of $(U, V)$ that follow not only the marginal empirical distributions $\widehat{F}_N$ and $F_N$, but also the empirical *joint* distribution of the residuals and the true errors. Then:

$$\begin{aligned} d_2\left(\widehat{F}_N, F_N\right)^2 &:= \inf_{\substack{U \sim \widehat{F}_N \\ V \sim F_N}} E\left[||U - V||^2\right] \\ &\leq E\Big[||U^* - V^*||^2\Big] \end{aligned}$$

because $(U^*, V^*)$ represents a choice of a single element from the set over which the infimum is taken. Expressing the right-hand side as the expectation of the joint empirical cumulative

distribution function:

$$
\begin{aligned}
&= \frac{1}{N}\sum_{n=1}^{N}\underbrace{\sum_{w=1}^{W}\left(\widehat{\epsilon}_{nw}-\epsilon_{nw}\right)^2}_{||\cdot||^2 \text{ of a } W\text{-vector}} \\
&= \frac{1}{N}\sum_{w=1}^{W}\underbrace{\sum_{n=1}^{N}\left(\widehat{\epsilon}_{nw}-\epsilon_{nw}\right)^2}_{||\cdot||^2 \text{ of an } N\text{-vector}} \\
&= \frac{1}{N}\sum_{w=1}^{W}||\widehat{\epsilon}_{w}-\epsilon_{w}||^2
\end{aligned}
$$

The interchange of summations is used to express $W$ norms involving residuals from different regressions, summed over $N$ observations, as $N$ norms involving residuals of observations within a regression, summed over $W$ regressions. Taking expectations and using Freedman (1981)'s Eq. (2.2), this implies:

$$
\begin{aligned}
E\Big[d_2\left(\widehat{F}_N, F_N\right)^2\Big] &= \frac{p}{N}\sum_{w=1}^{W}\sigma_w^2 \\
&\xrightarrow[N\to\infty]{} 0
\end{aligned}
$$

By Jensen's inequality:

$$
E\Big[d_2\left(\widehat{F}_N, F_N\right)\Big] \xrightarrow[N\to\infty]{} 0
$$

□

**Lemma 3.** *Regarding the the second term on the right-hand side of Equation (4), we have:*

$$
d_2\left(F_N, F\right) \xrightarrow[N\to\infty]{P} 0
$$

*Proof.* Letting $P_N$ denote an empirical probability, $F_N$ can be expressed as:

$$
\begin{aligned}
P_N\left(\epsilon_{n1}\le c_1,\cdots,\epsilon_{nW}\le c_W\right) &= \frac{1}{N}\sum_{n=1}^{N}1\Big\{\epsilon_{n1}\le c_1,\cdots,\epsilon_{nW}\le c_W\Big\} \\
&\xrightarrow[N\to\infty]{A.S.} P\left(\epsilon_{n1}\le c_1,\cdots,\epsilon_{nW}\le c_W\right)
\end{aligned}
$$

with the last line following from the Strong Law of Large Numbers (SLLN). Thus, $F_N \xrightarrow[N\to\infty]{A.S.} F$. Also by the SLLN, $\int ||x||^p \; F_N(dx) \xrightarrow[N\to\infty]{A.S.} \int ||x||^p \; F(dx)$ because the left-hand side is a sample average whereas the right-hand side is its expectation. These two results immediately imply Condition (a) of Bickel & Freedman (1981)'s Lemma 8.3, which yields $d_2\left(F_N, F\right)^2 \xrightarrow[N\to\infty]{P} 0$ and hence the desired result. □

**Theorem 4.** *The residual bootstrap is weakly consistent under the Mallows-Wasserstein metric for the OLS coefficient estimates (Definition 29.2 of DasGupta (2008)); that is:*

$$d_2\left(\Psi(F), \Psi(\widehat{F}_N)\right) \xrightarrow[N\to\infty]{P} 0$$

*Proof.* Combining Theorem 3 with the triangle inequality in Equation (4) and observing that $\text{tr}\{BB'\} = \sum_{n=1}^{N} B_N^2 \geq 0$ yields:

$$d_2\left(\Psi(F), \Psi(\widehat{F}_N)\right) \leq \sqrt{N \cdot \text{tr}\{BB'\}} \cdot \left(d_2\left(\widehat{F}_N, F_N\right) + d_2\left(F_N, F\right)\right)$$

The term $\sqrt{N \cdot \text{tr}\{BB'\}} \xrightarrow[N\to\infty]{P} k$ by Assumption 2 because $BB'$ is scalar. By Markov's inequality, the convergence in mean of Lemma 2 implies that $d_2\left(\widehat{F}_N, F_N\right) \xrightarrow[N\to\infty]{P} 0$. Last, by Lemma 3, $d_2\left(F_N, F\right) \xrightarrow[N\to\infty]{P} 0$, so the desired result holds. □

The next theorem uses the above result regarding convergence of the resampling-based coefficient estimates to establish convergence of the test statistics.

**Theorem 5.** *Algorithm 1 fulfills Assumption 1 (main text); namely:*

$$T^{(j)} \xrightarrow[N\to\infty]{D} T^0$$

*Proof.* By Bickel & Freedman (1981)'s Lemma 8.3, Theorem 4 implies that

$$\sqrt{N}\left(\widehat{\beta}^{W(j)} - \widehat{\beta}^{W}\right) \xrightarrow[N\to\infty]{D} \sqrt{N}\left(\widehat{\beta}^{W} - \beta^{W}\right)$$

By Freedman (1981)'s Theorem 2.2, each $\widehat{\sigma}_w^{(j)} \xrightarrow[N\to\infty]{P} \sigma_w$. The desired result then follows from the multivariate Slutsky's Theorem. □

## 3. Proof of Theorem 1

Define the $r$-family of "rejection sets" as all possible configurations of the $W$ test statistics that lead to $r$ rejections:

$$\mathcal{A}_r = \Big\{ (A_1, \cdots, A_W) \in \mathbb{R}^W : (T_1 \in A_1, \cdots, T_W \in A_W) \;\Rightarrow\; \hat{\theta} = r \Big\}$$

Consider the limiting distribution of $\hat{\theta}^{(j)}$:

$$\begin{aligned}
\lim_{N\to\infty} P\left(\hat{\theta}^{(j)} = r\right) &= \lim_{N\to\infty} P\left(\sum_{w=1}^{W} 1\left\{T_w^{(j)} > c_{w,\alpha}\right\} = r\right) \\
&= \lim_{N\to\infty} \sum_{(A_1,\cdots,A_W)\in\mathcal{A}} P\left(T_1^{(j)} \in A_1, \cdots, T_W^{(j)} \in A_W\right) \\
&= \sum_{(A_1,\cdots,A_W)\in\mathcal{A}} P\left(T_1^0 \in A_1, \cdots, T_W^0 \in A_W\right) \\
&= P\left(\hat{\theta}^0 = r\right)
\end{aligned}$$

where the second equality follows from Assumption 1 (main text).

## 4. Proof of Theorem 2

The probability of obtaining more than $f$ false positives across tests at level $\alpha$ is:

$$P\left(\sum_{w\in\mathcal{K}'} 1\{T_w > c_{w,\alpha}\} > f \,\middle|\, H_{0,w} \text{ holds for exactly } w \in \mathcal{K}'\right)$$

By subset pivotality, the conditioning statement can be expanded to include the hypotheses outside $\mathcal{K}'$, such that the global null holds:

$$= P\left(\sum_{w\in\mathcal{K}'} 1\{T_w > c_{w,\alpha}\} > f \,\middle|\, H_{0,w} \text{ holds } \forall\, w \in \mathcal{W}\right)$$

Conditional on the global null, we have $\mathcal{K}' = \mathcal{W}$; hence:

$$\begin{aligned}
&= P\left(\sum_{w\in\mathcal{W}} 1\{T_w > c_{w,\alpha}\} > f \,\middle|\, H_{0,w} \text{ holds } \forall\, w \in \mathcal{W}\right) \\
&= P\left(\hat{\theta} > f \,\middle|\, H_{0,w} \text{ holds } \forall\, w \in \mathcal{W}\right) \\
&= P\left(\hat{\theta}^0 > f\right)
\end{aligned}$$

Choosing $f = \theta_{hi}$, the above equality becomes:

$$\begin{aligned}
P\left(\sum_{w\in\mathcal{K}'} 1\{T_w > c_{w,\alpha}\} > \theta_{hi} \,\middle|\, H_{0,w} \text{ holds } \forall\, w \in \mathcal{K}'\right) &= P\left(\hat{\theta}^0 - \theta_{hi} > 0\right) \\
&\lesssim 0.05
\end{aligned}$$

The asymptotic bound holds by Theorem 1 in the main text. The left-hand side represents the probability of obtaining more than $\theta_{hi}$ false positives under any configuration of true and false hypotheses, $(H_{0,1}, \cdots, H_{0,W})$, because $\mathcal{K}'$ is simply the arbitrary subset of $\mathcal{W}$ for which $H_{0,w}$ does hold. The right-hand side represents the probability of observing at least one excess hit.

## 5. Additional results for the applied example

Table S1 displays demographic and childhood family characteristics of the analyzed sample.

**Table S1:** *Demographic and childhood family characteristics of 2,697 analyzed subjects. [a]: By subject's adolescence, subject's family had ever been on welfare. [b]: Ranged from 1 ("a lot better off" than others) to 7 ("a lot worse off" than others). [c]: By age 16, subject had ever lived with an alcoholic.*

| Characteristic | Mean (SD) or % |
|---|---|
| Age | 46.89 (12.35) |
| Female | 53.7% |
| Race | |
| White | 93.3% |
| Black | 3.6% |
| Other | 3.2% |
| Born in US | 95.8% |
| Mother born in US | 90.5% |
| Father born in US | 90.2% |
| Lived with biological parents | 81.1% |
| Number of siblings | 2.92 (1.57) |
| Highest parental education | |
| Less than high school | 25.8% |
| High school | 36.0% |
| Some college | 15.8% |
| College degree or more | 22.5% |
| Childhood welfare[a] | 5.6% |
| Subjective SES[b] | 4.07 (1.29) |
| Residential area | |
| Rural | 23.1% |
| Small town | 25.6% |
| Medium town | 12.1% |
| Suburbs | 16.8% |
| City | 18.3% |
| Moved around | 4.1% |
| Residentially stable | 74.1% |
| Mother smoked | 32.6% |
| Father smoked | 62.0% |
| Lived with alcoholics[c] | 20.9% |
| Importance of religion | |

*Continued on next page*

Table S1 – *continued*

| Characteristic | Mean (SD) or % |
| --- | --- |
| Very important | 43.5% |
| Somewhat important | 35.7% |
| Not very important | 16.0% |
| Not at all important | 4.7% |

# 6. Additional simulation results

The following figures show additional scenarios for scenarios with $W = 40$ outcomes.

**Figure S1:** *95% null intervals versus mean rejections in observed datasets ($\times$). Panels: Null and alternative data-generating mechanisms of original samples. Points and error bars: Mean $\widehat{\theta}^{(j)}$ and mean limits of null intervals with tests at $\alpha = 0.01$ (yellow) or at $\alpha = 0.05$ (red).*

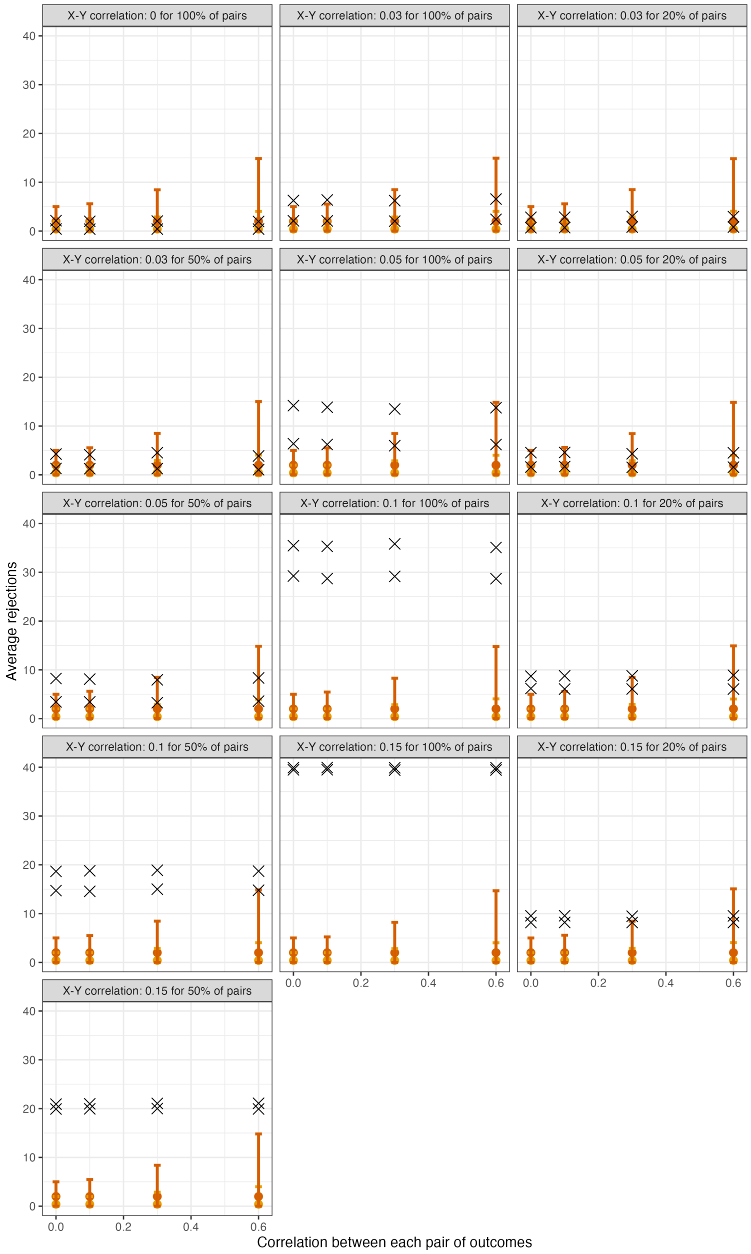

**Figure S2:** *Power of global tests based on existing FWER-control procedures and on the number of rejections. "Global (alpha=0.01)" and "Global (alpha=0.05)": proposed methods. The final panel represents Type I error under the global null.*

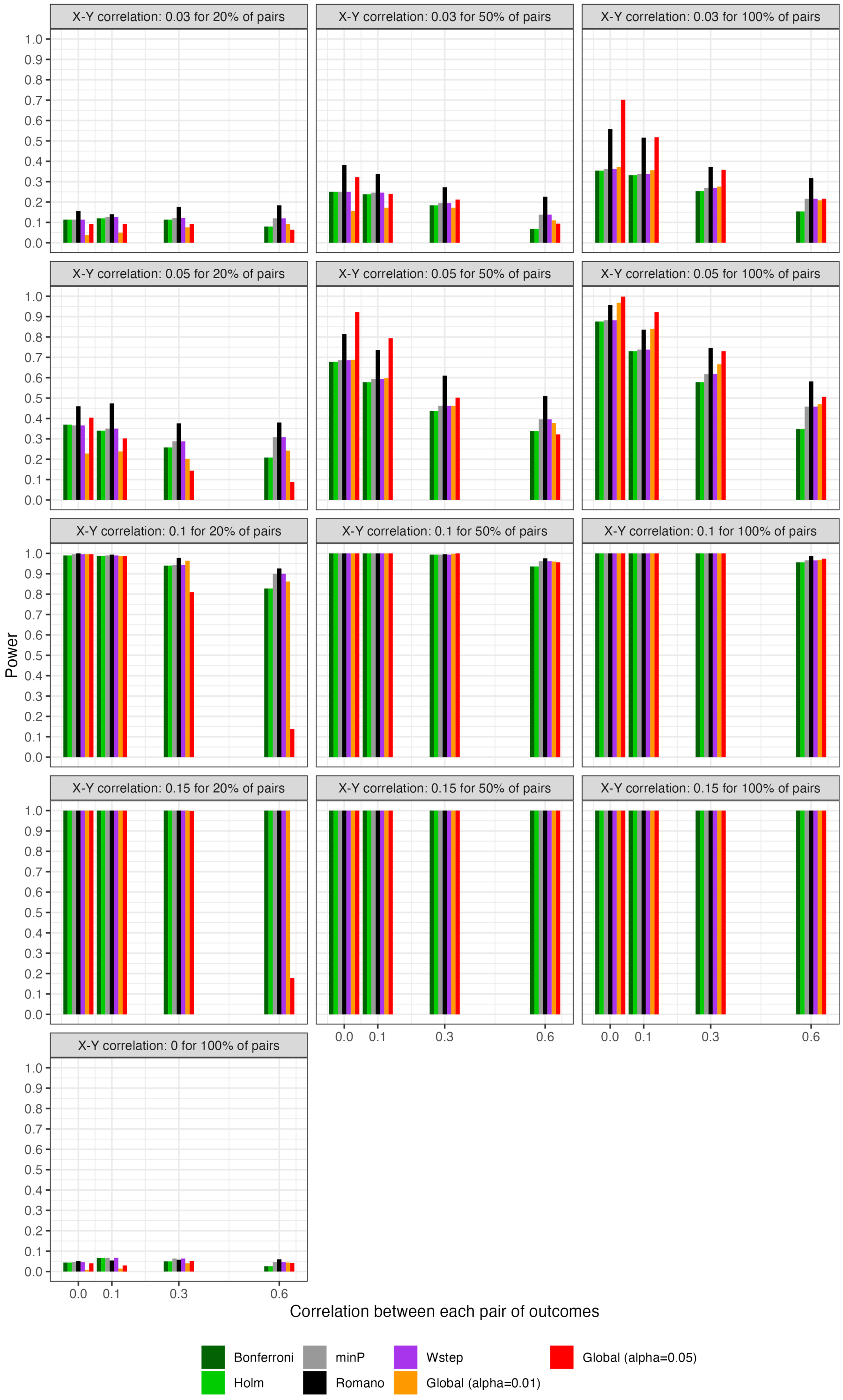

**Figure S3:** *Number of rejected null hypotheses at familywise-controlled* $\alpha_W = 0.05$ *based on existing FWER-control procedures and on the excess hits. "Global (alpha=0.01)" and "Global (alpha=0.05)": proposed methods. Red dashed line: Actual number of false null hypotheses* $(q \times W)$.

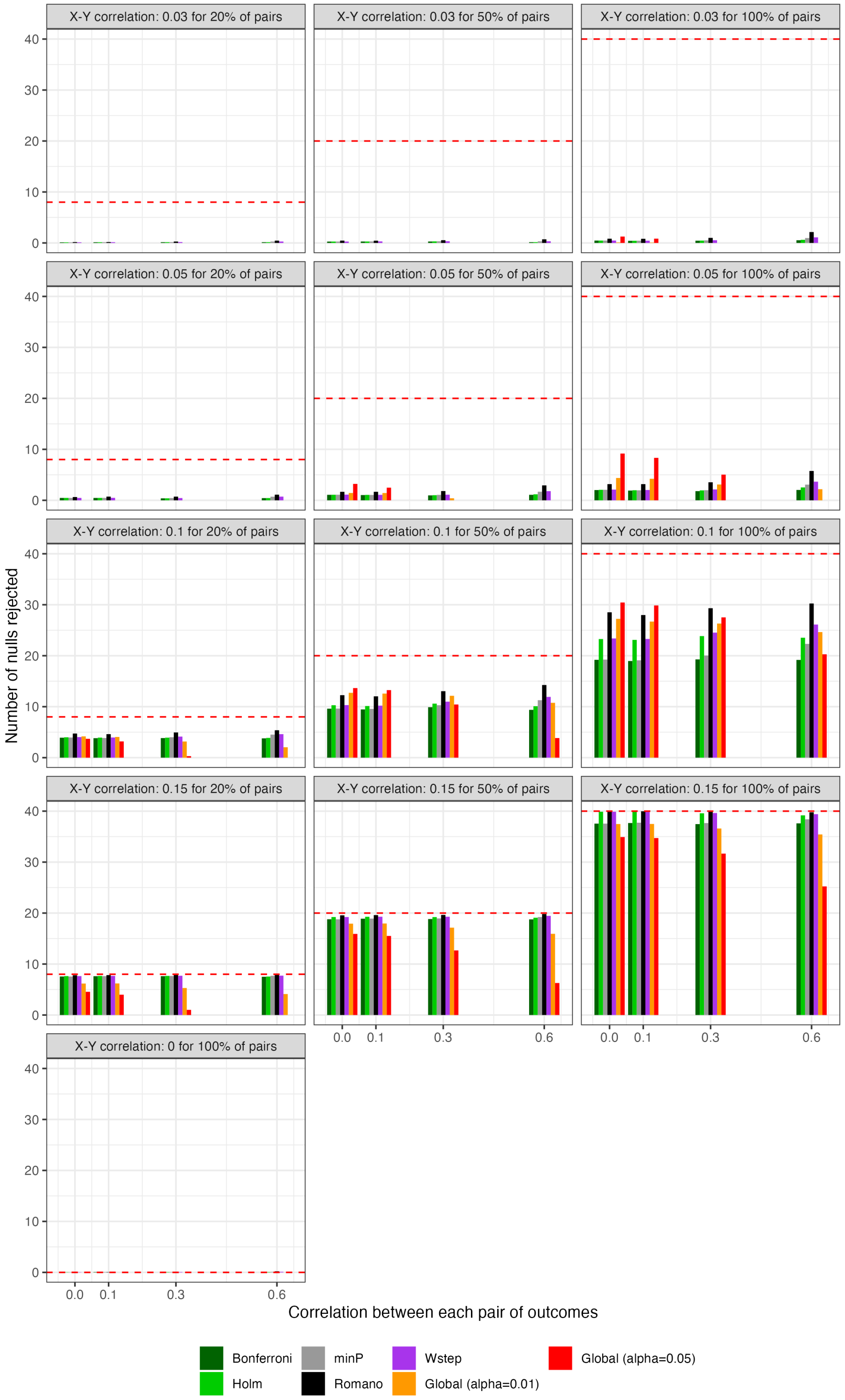

## 6.1. Comparison of $p$-values adjusted by existing methods

We performed a rudimentary visual comparison of $p$-value adjustments produced by one naïve method (Holm) and one resampling-based method (Wstep). We generated a single dataset as in the simulation study with 1 covariate, 100 outcomes, $N = 1,000$, $\rho_{XY} = 0.08$ for all outcomes, and $\rho_{YY} = 0.25$. We chose these parameters to yield a large number of adjusted $p$-values $< 0.05$ for illustrative purposes. Figure S4 plots the 100 $p$-values adjusted using the Holm and Wstep methods (obtained by resampling as in the applied example with $B = 500$ resamples) and suggests that in this simple simulation, the methods differ little in their adjustments to $p$-values near $\alpha = 0.05$; rather, the differences appear to emerge primarily for $p >> 0.05$. We obtained qualitatively similar results when comparing other pairs of existing methods (not shown).

**Figure S4:** *$p$-values in a single simulated dataset adjusted by the Holm method versus the Wstep method. Red dashed lines: $\alpha = 0.05$ threshold.*

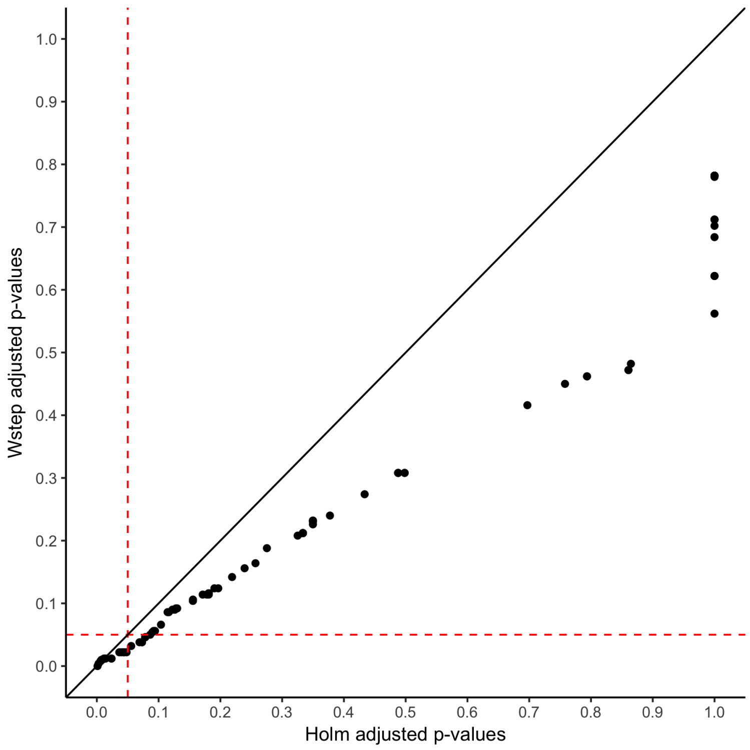


# 7. Introduction to the package NRejections

Here we briefly describe the `R` package `NRejections`; note that additional functions, details, and additional examples are available in the standard `R` documentation. For OLS models as described in Section 4.2 of the main text, the null interval, excess hits, and global test can be conducted by calling a single wrapper function, `corr_tests`. This function first fits the $W$ models in the original dataset, adjusting for any user-specified covariates. Then, resamples

are generated via Algorithm 1 (main text) and used to estimate and return our proposed metrics, along with estimates and inference from the original sample. Optionally, the global test can additionally be conducted using any combination of methods in Table 1 (main text). Below is a minimal example.

```
# this was run on R version 3.3.3
# and NRejections version 1.0.0

library(NRejections)

# simulate data with 40 outcomes and 1 covariate of interest,
#  similarly to simulation study
# 80% of the 40 associations are non-null (correlation strength of 0.08);
#  and the others are null
cor = make_corr_mat( nX = 1,
  nY = 40,
  rho.XX = 0,
  rho.YY = 0.15,
  rho.XY = 0.08,f
  prop.corr = .8 )

d = sim_data( n = 1000, cor = cor )

# may take 5-10 min to run on 8-core personal computer
res = corr_tests( d,
                  X = "X1",
                  Ys = names(d)[ grep( "Y", names(d) ) ],
                  B = 1000,
                  method = "nreject" )

# main results
res$null.int
res$excess.hits
res$global.test
```

As described in the Discussion, Algorithm 1 is more broadly applicable to multiple-testing procedures outside the scope of this paper. For these general applications, the user could first obtain residuals and point estimates from the original dataset using the function `dataset_result` and pass these to `resid_resample`, which returns matrices of $p$-values and test statistics from the resamples. See `?resid_resample` for examples.

# References

Bickel, P. J., & Freedman, D. A. (1981). Some asymptotic theory for the bootstrap. *The Annals of Statistics*, 1196–1217.

DasGupta, A. (2008). *Asymptotic theory of statistics and probability.* Springer, New York.

Freedman, D. A. (1981). Bootstrapping regression models. *The Annals of Statistics*, 1218–1228.
